# A bioinspired gelatin-hyaluronic acid-based hybrid interpenetrating network for the enhancement of retinal ganglion cells replacement therapy

**DOI:** 10.1038/s41536-021-00195-3

**Published:** 2021-12-20

**Authors:** Pierre C. Dromel, Deepti Singh, Eliot Andres, Molly Likes, Motoichi Kurisawa, Alfredo Alexander-Katz, Myron Spector, Michael Young

**Affiliations:** 1 Massachusetts Institute of Technology, Cambridge, MA USA; 2 Schepens Eye Research Institute of Massachusetts Eye and Ear, Harvard Medical School, Boston, MA USA; 3 Wellesley College, Wellesley, MA USA; 4 A*STAR Institute of Bioengineering and Nanotechnology, Singapore, Singapore; 5 VA Boston Healthcare System, Brigham and Women’s Hospital, Harvard Medical School, Boston, MA USA

**Keywords:** Regenerative medicine, Neural stem cells, Retina, Biomaterials - cells, Tissue engineering

## Abstract

Biomaterial-based cell replacement approaches to regenerative medicine are emerging as promising treatments for a wide array of profound clinical problems. Here we report an interpenetrating polymer network (IPN) composed of gelatin-hydroxyphenyl propionic acid and hyaluronic acid tyramine that is able to enhance intravitreal retinal cell therapy. By tuning our bioinspired hydrogel to mimic the vitreous chemical composition and mechanical characteristics we were able to improve in vitro and in vivo viability of human retinal ganglion cells (hRGC) incorporated into the IPN. In vivo vitreal injections of cell-bearing IPN in rats showed extensive attachment to the inner limiting membrane of the retina, improving with hydrogels stiffness. Engrafted hRGC displayed signs of regenerating processes along the optic nerve. Of note was the decrease in the immune cell response to hRGC delivered in the gel. The findings compel further translation of the gelatin-hyaluronic acid IPN for intravitreal cell therapy.

## Introduction

Cell replacement therapies critically depend on: the viability of the cells being delivered; maintenance of their phenotype; and their engraftment in the targeted tissue^1,2^. Many cell therapies have failed to achieve desired outcomes, partly due to low cell viability and failure to attach to the site of injury or disease^3^. In most cases, the exogenous cells are delivered to the target site in a buffered saline solution, raising questions: are the cells receiving the necessary physical and chemical stimuli; and are the cells being retained at the target site long enough for them to bind to the host cells or extracellular matrix to become integrated into the tissue (i.e., engraft)?^4^

For ophthalmologic applications, biomaterial replacements for saline need to be injectable and be able to undergo transformation to a solid material once in vivo. While a host of hydrogels can meet these requirements, it is only a few that can undergo spontaneous covalent cross-linking in situ in a way that enables the control of gelation, degradation rate, and mechanical behavior^5^. One such polymer proven to be able to do so is a gelatin conjugate with hydroxyphenyl propionic acid (Gtn-HPA). In addition to its advantage being able to undergo enzyme-mediated covalent cross-linking, it provides cell adhesion ligands, protects cells from stress exerted during transplantation and undergoes enzymatic degradation in vivo. Moreover, one recent study demonstrated substantial increases in viability and proliferation, and maintenance of phenotype, of retinal progenitor cells injected through a 31-gage needle when they were incorporated in Gtn-HPA compared to saline, owing to the protection of the cells from the imparted shear stress offered by the gel^6,7^.

Several neurodegenerative diseases including Glaucoma, a leading cause of blindness^8^, result in a progressive loss of retinal ganglion cells (RGC) requiring an investigation of the intravitreal injection of neuro-modulatory cells^9^ or RGC^10,11^. While all cell replacement therapies, require the exogenous cells to migrate from the delivery site to their natural location within the host, RGC replacement therapy faces special challenges^12,13^: intravitreally delivered RGC need to penetrate the inner limiting membrane (ILM), extend afferent processes to connect with bipolar and amacrine cells; and extend efferent processes (axons) to connect with specific targets in the brain. The migration of exogenous RGCs through the ILM into the ganglion cell layer and their adhesion to other retinal and brain cells is dependent on the cell adhesion molecules that they express.

Prior studies (including gene therapies^14^ and cell transplantations^15^) have shown that the ILM is a major barrier to the retinal integration of exogenous RGCs. The ILM is a basement membrane mainly composed of collagen IV, laminin, and others ECM proteins^16^. Therefore, cellular interactions of injected RGC with the ILM play a major role in their integration. Other studies have shown that reactive gliosis, rather the the ILM, may be the reason for engraftment failure of injected RGCs in the vitreous^12,17^. Overall, the interactions between exogenous cells and the proteins present in the ILM determine the possibilities of engraftment, spatial patterning, and possible cell replacement of RGCs^18^.

Transplantation of RGC into the vitreous, a large Interpenetrating network made of collagen fibers and hyaluronic acid^19^, could be regulated by an injectable polymeric network. The molecular framework of a composite gel could provide adhesion ligands for cell integrins^20^, and thereby affect CAM expression^21^. Having the modulus compatible with that of the tissue into which it is injected could also affect CAM expression, as well as facilitate attachment of the gel to tissue structures: e.g., to the ILM of the retina.

Toward those ends, we formulated a gel comprising Gtn-HPA and hyaluronic acid tyramine (HA). In order to enable enzyme-induced covalent crosslinking of HA, it was conjugated with tyramine (Tyr)^22^. Horseradish peroxidase and hydrogen peroxide incorporated into the gel enabled the independent control of gelation rate and cross-link density of Gtn-HPA/HA-Tyr (Gtn-HA) formulations.

Here we show that by designing a hybrid IPN suitable for vitreal injection, we are able to transplant, grow and engraft hRGC, which eventually display signs of regenerating processes along the optical nerve. By precisely matching the chemical and mechanical properties of our hydrogel with the vitreous we achieved attachment to the retina (overcoming the ILM barrier) and enhanced cell migration, engraftment, and retinal regeneration.

Our work will be useful for regenerating vision in patients suffering from retinal diseases, but more generally, the materials design principles herewith put forward should help other stem cell therapies.

## Results

### IPN tunability properties and characterization

**Fig. 1:**
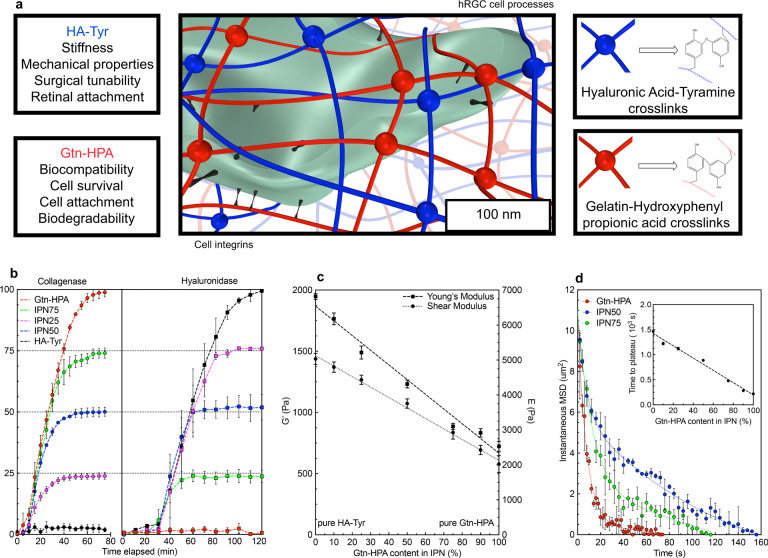
The Gelatin and Hyaluronic Acid interpenetrating network system and its mechanical and chemical characterization. **a** Schematic and 3D model of a human cell encapsulated in the IPN made of Gelatin-HPA (red) and HA-Tyr (blue) with integrins bonding to Gtn-HPA only (black). Legends shows the two different crosslinks with their respective chemical structures. **b** Hydrogels’ degradation assays performed with Collagenase and Hyaluronidase treatment for Gtn-HPA (red), IPN75 (green), IPN50 (blue), IPN25 (pink) and HA-Tyr (black). Degradation assay, comprising *n* = 15 replicates, was measured every 5 min with collagenase and every 10 min with Hyaluronidase. **c** Shear (dot line) and Young’s (dash line) moduli measurements for multiple IPN with different Gtn-HPA content (ranging from 10% to 90%). Linear regression was applied both set of values, with *n* = 15. **d** Mean square displacement measurements of PLGA microbeads encapsulated in materials during microrheology assay for Gtn-HPA (red), IPN75 (green) and IPN50 (blue). *N* = 15 microbeads were tracked for each sample and the data were fitted with a double exponential decay regression. Inset (**d**) shows the time to reach stability of the different IPN during the gelation process. Linear regression was applied to the data confirming the increase of gelation time with the addition of HA-Tyr in the mix. All data is shown as mean ± SEM.

The development of a bioinspired gel with suitable characteristics for intravitreal injection (Fig. 1a) was initiated by an evaluation of the viability of hRGC in gelatin-based gels with increasing concentrations of HA. Our results suggest that networks incorporating Gtn-HPA, and HA-Tyr have a superior biocompatibility (reflected in cell viability) and can be considered as candidate for retinal cell replacement therapy (see Supplementary Fig. 1).

For our system, both the Gtn and HA networks are in-situ enzymatically crosslinked with horseradish peroxidase (HRP) and H_2_O_2_ (see details in Methods)^23^. Thus, our first goal was to explore if there was any observable selectivity in the crosslinking. If there was, we expected to obtain a hybrid interpenetrating network (IPN) instead of a random heteropolymer network. Our results showed that the crosslinking is very specific to the respective polymer. To prove this, we prepared networks with varying percentages of Gtn-HPA and HA-Tyr (from 100% Gtn-HPA to 100% HA-Tyr) and enzymatically degraded them with collagenase and hyaluronidase^24^. As seen in Fig. 1b, applying either collagenase or hyaluronidase treatment degrades the exact proportion of Gtn-HPA or HA-Tyr, respectively, that was used in the preparation of network: e.g. IPN75 (containing 75% of Gtn-HPA and 25% of HA-Tyr) demonstrated a mass loss of 75% when mixed with collagenase and a mass loss of 25% when mixed with hyaluronidase. Complete degradation was observed on homopolymeric networks combined with their respective enzymes. This, along with the Fourier transform infrared spectroscopy (Supplementary Figure 2), confirms the formation of a hybrid IPN.

To assess whether the mechanical tunability of these IPN can be controlled by their relative percentages of Gtn and HA, we performed oscillatory rheology and unconfined compression testing (Supplementary Fig. 3). As seen in Fig. 1c, the shear (G’) and Young’s (E) modulus decrease monotonically with the increasing amount of Gtn-HPA in the IPN, ranging respectively from 1438 Pa and 6828 Pa for pure HA-Tyr to 578 Pa and 2532 Pa for pure Gtn-HPA. This behavior can be explained by homopolymeric networks rheology (Supplementary Fig. 3) which showed that HA-Tyr possesses a shear modulus 2–5 times higher than Gtn-HPA. Mechanical and stiffness tunability can therefore be controlled by the IPN content of the respective polymers.

For in-situ crosslinking hydrogels, controlling the gel point is critical in terms of surgical performance: to avoid needle clogging. To measure the gel points of our samples we employed microrheology to deal with the extremely fast gelation of these IPN. As explained in Methods, PLGA microbeads were mixed with the networks and their instantaneous mean square displacement (MSD), due to Brownian motion, was measured over time (see Supplementary Movie 1, Supplementary Movie 2, and Supplementary Movie 3). While all samples (Gtn-HPA, IPN75, and IPN50) demonstrated a double exponential decay of microbeads MSD (Fig. 1d), a positive correlation was found between the gel point time and the HA-Tyr content of the IPN. Supplementary Figure 3 shows the gel points ranging from 42 s for Gtn-HPA to 2min47s for IPN50, showing that gelation time was tunable by controlling the Gtn-HPA content of IPN. By measuring the time for G’ to reach the plateau in oscillatory rheology (inset in Fig. 1d) we could detect the time required for hydrogels to reach steady-state equilibrium (stability of IPN), which increased with the percentage of HA-Tyr.

Complete characterization of solid polymers (Supplementary Figure 4), homopolymeric networks, and IPN was performed and is summarized in Table 1. These findings enable us to consider these hybrid IPN, made of Gtn-HPA and HA-Tyr, potential candidates for cell encapsulation, in vivo injection and enhancement of retinal regeneration.

**Table 1:** Complete characterization of solid polymers and wet gels.

	Polymer (wt %)	G’ (Pa)	E (Pa)	Gel Point (s)	Time to plateau (s)	Mn (kg/mol)	Mw (g/mol)	Tg (C)	Tm (C)
**Gtn-HPA**	2	578 ± 32	2532 ± 20	42.5	220 ± 18	18,000 ± 40	64,500 ± 75	140 ± 1	167 ± 2
**IPN75**	2	835 ± 26	3101 ± 24	151	479 ± 10				
**IPN50**	2	1072 ± 29	4316 ± 39	167	888 ± 9				
**HA-Tyr**	2	1438 ± 13	6818 ± 76	273	1463 ± 5	68,000 ± 55	153,70 ± 20	148 ± 2	188 ± 1

Data measured with oscillatory rheology, unconfined compression testing, microrheology, gel permeation chromatography, and differential scanning calorimetry. Data shown as mean + /- SEM for *n* = 5 replicates.

### Optimal IPN for cell encapsulation and in vivo injection

**Fig. 2:**
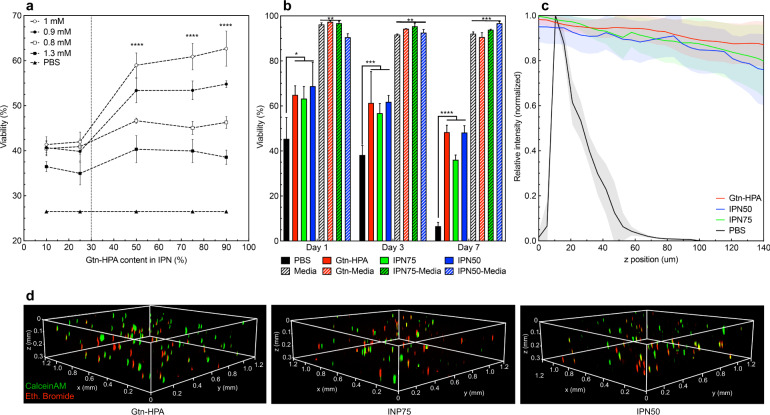
Optimal interpenetrating network candidates for human retinal ganglion cells viability, encapsulation, and in vivo release. **a** Viability assay at day 3 of encapsulated human retinal ganglion cells in IPN with different Gtn-HPA content and a range of crosslinker concentration compared with PBS. Data were measured for 10 field of views of live/dead staining. Data shown as mean ± SEM and one-way ANOVA followed by student *t* test was performed showing a statistically high significant difference between H_2_O_2_-1mM and all others for more than 50% Gtn-HPA in the IPN (****p < 0.0001). **b** Viability assay through the time of encapsulated hRGC in hydrogel candidates (Gtn-HPA, IPN75, and IPN50) with media or PBS. Plain bars show cells deprived from nutrients while hashed bars represent cells in their defined medium. Data shown as mean ± SEM of triplicate wells with 15 different fields. Two-way ANOVA, followed by Tukey’s test, was performed and shows a statistically high significant difference between samples including media and all other groups (**p = 0.005 and ***p = 0.0001) at all time points; and between hydrogels groups deprived from nutrients compared to PBS (*p = 0.01 and ****p < 0.0001). **c** Relative fluorescence intensity of hRGC stained with live/dead assay, imaged with confocal microscopy for Gtn-HPA, IPN75, IPN50, and PBS, on 150 um thick slides, in the function of their z position. d. 3D fluorescence image of live (CalceinAM-green) and dead (Ethidium Bromide-red) hRGC encapsulated in the hydrogel candidates, imaged with confocal microscopy on 1.2×1.2×0.3 mm samples. All images were taken at 10X magnification under fluorescence microscopy.

Using hydrogels for cell encapsulation implies tuning the materials to be able to conserve many cell characteristics: high viability, differentiation, phenotype, shape, and distribution through the gel. For this investigation, hRGC were encapsulated in multiple Gtn-HA IPN gels with selected percentages of Gtn and HA, and various crosslinker concentrations. A short-term viability study, seen in Supplementary Fig. 5 and Fig. 2a, enabled us to find the optimal crosslinker (H_2_0_2_) concentration both in IPN and homopolymeric hydrogels: 1 mM. In Fig. 2a, we also studied the impact of Gtn-HPA content in IPN on encapsulated cell viability. A viability threshold, corresponding to a content of >30% of Gtn-HPA, was observed for all crosslinker concentrations demonstrating that hRGC do not thrive in HA-Tyr. This finding can be related to the understanding that hRGC possess integrins to attach to the gelatin backbone but not to the hyaluronic acid backbone^20,25^. This result reduces the number of candidates to IPN possessing a Gtn-HPA content higher than 50% (which include Gtn-HPA itself).

To compare the efficacy of various IPN formulations, we proceeded to in vitro and in vivo experiments with Gtn-HPA, IPN75, and IPN50. In vitro viability assay was performed (1,3, and 7 days) by leaving cells in PBS or adding defined medium in hydrogels (Fig. 2b). hRGC receiving medium, were significantly more viable than those in monolayer receiving PBS, even after 7 days, suggesting a favorable diffusion of nutrients through the IPN. However, when cells received no nutrients viability decreased significantly with time, reaching <7% for the PBS sample after 7 days. Notably, IPN reduced this drop of viability by protecting the cells from the lack of nutrients for all time points, being significantly higher for all IPN groups compared to PBS samples (35–45% viable after 1 week of medium deprivation). This result^26^ suggests that these IPN are compelling candidates for enhancing the ability of encapsulated cells to contribute to the restoration of retinal function.

To determine the distribution of cells throughout hydrogels, the intensity of live (CalceinAM-FITC) and dead (Ethidium Bromide-APC) cells was averaged, normalized, and measured as a function of location (cubic regions: 150 um on a side), Fig. 2c. Overall, more than 75% of each sample of analyzed to reduce the selectivity of ROIs during quantification. The microscope slide samples displayed a peak in intensity around its zero position with a quickly decaying intensity while all hydrogel samples show a constant intensity in all directions. This suggests a uniform distribution of both live and dead cells inside the IPN, which is critical for the constant release of cells at the injection site due to the enzymatic and surface degradation of our in situ-crosslinking hydrogels.

Confocal microscopy enabled the visualization of the size, morphology, and distribution of hRGC (after live/dead staining) throughout 300 um thick sections of the gels (Fig. 2d). Image processing algorithms (Supplementary Fig. 6) permitted the quantification of live cell size and shape factor in all samples. hRGC in the gels were significantly larger compared to 2D, and their shape factor was lower reflecting a more elongated morphology (Supplementary Fig. 7). This finding suggests that hydrogels may enable certain cell behavior, including the formation of cell processes^27^, which are not seen with cells cultured in monolayer in PBS or medium.

Cell phenotype was checked by flow cytometry (gating strategy presented in Supplementary Figure 8) and immunohistochemistry after 7 days of culture in the medium in 2D or 3D (gels) environments. Phenotype results, as seen in Supplementary Figure 9, were consistent with previous studies^28^ with no significant difference in proliferation, apoptosis, or stemness markers between groups. Our results advocate for the use of IPN50, IPN75, and Gtn-HPA for in vivo transplantation and injection of hRGC.

### In vivo attachment of IPN to the retina

**Fig. 3:**
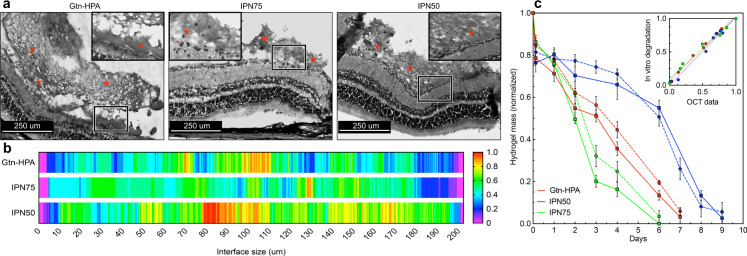
In vivo proof of attachment of hydrogel candidates to the inner limiting membrane (ILM) of the retina. **a** Hematoxylin and Eosin (H&E) staining of rat’s retina 3 days post hydrogel injections for Gtn-HPA, IPN75, and IPN50 groups. Images were taken under bright-field microscopy at 10X and 63X magnification for respectively large image and inset. Hydrogel presence is observed and labelled with red stars. Inset shows higher magnification and interface of gel with ILM to prove attachment. The scale bar is 250 um. **b**. Analysis of hydrogel-ILM interface from H&E staining with heat map. 200 um length interfaces were analyzed for all three samples. Heat map ranges from 0 with no attachment at the interface to 1 with attachment seen on all different slides. **c**. Gtn-HPA, IPN75, and IPN50 hydrogels mass degraded through time during in vivo injection compared with in vitro degradation. In vitro degradation (plain lines) was performed using concentrations of collagenase and hyaluronidase found in the eye^30,31^, while in vivo degradation was measured using SD-OCT and image processing (for more information see Method and Supplementary Fig. 10). Normalized hydrogel mass was measured for *N* = 15 replicates every 1–2 days. The correlation coefficient between in vitro and in vivo was measured and are shown in **inset**. *R*^2^ = 0.97 was found for all samples with no statistical difference found between in vivo and in vitro data. Data are shown as mean ± SEM.

Due to the viscous nature of the vitreous, vitreal injections of retinal stem/progenitor cells or drugs pose certain problems, including how to control their location and release onto the retina? To begin to investigate whether our gels can enhance intravitreal cell therapy, we injected Gtn-HPA, IPN50, and IPN75 (with no cells) into the vitreous of Long Evans rats and assessed, noninvasively, daily changes in their vitreous and retina using SD-OCT. At sacrifice, 3 days postinjection, we evaluated the retina histology with H&E staining (Fig. 3a). Histological images showed the presence of islands of gels present in the vitreous and attached to the inner limiting membrane (ILM) of the retina^29^. By incorporating the hRGC into these Gtn-HA gels, a controlled and constant release of the cells to the ILM could be achieved. Of note, no detachment or injury to the retina was observed throughout the experiment suggesting that no harm was caused to the retina during the injection of gels.

To quantify this finding, we analyzed the interfaces between the gels and retina (Supplementary Fig. 10). This analysis indicated an increasing average percent of attachment with increasing HA content and stiffness: 50% for Gtn-HPA, 59% for IPN75, and 79% for IPN50. The attachment was measured for *n* = 10 and normalized per 200 um portions of the ILM, which reflected the holes at the interface and therefore the capability of each gel to attach to the ILM. As seen in Fig. 3b, IPN50 displayed the highest degree of attachment with the lowest number of holes, while Gtn-HPA distribution along the ILM was uneven with numerous holes along the interface.

Finally, SD-OCT data (Supplementary Fig. 11) were analyzed to provide an approximation of the amount of gel present in the vitreous (or on top of the retina) with time (see Methods) and was compared with an in vitro degradation assay of IPN using collagenase and hyaluronidase (enzymes present in the eye^30,31^). As seen in Fig. 3c, all IPN degraded in vivo with time with the longest degradation time for IPN50 after 9–10 days. In vitro degradation showed a comparable (see inset Fig. 3c with averaged *R*^2^ = 0.97) result which confirms the presence of enzymes in vivo with corresponding concentrations used in vitro (Methods). A curious trend is shown for IPN75 degrading faster than Gtn-HPA besides being stiffer. This can be explained by the presence of both hyaluronidase and collagenase in the vitreous. IPN75, comprising 75% Gtn-HPA, and 25% HA-Tyr, will be affected by both collagenase and hyaluronidase while the homopolymeric hydrogel, Gtn-HPA, will just be degraded by collagenase only (as we proved previously with in vitro degradation).

Being able to attach our in situ-crosslinking hydrogel to the back of the eye (ILM) is one of the core results of our study underscoring its ability to enhance not only cell therapies but also vitreal drug injections^32^.

### In vivo vitreal injection of encapsulated hRGC in IPN

**Fig. 4:**
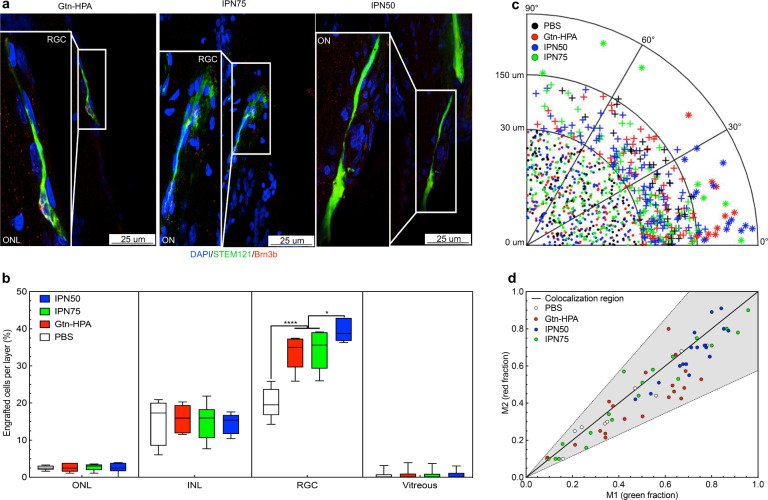
In vivo vitreal injection of encapsulated human retinal ganglion cells in Long Evans rats. **a** Fluorescence microscopy images of retina sections 1-month post transplantation. All images were taken at 63X magnification with confocal microscopy (scale bar – 25 um). Slides were stained with DAPI-VioBlue (nuclei staining), STEM121-FITC (human marker), and Brn3b-TdTomato (RGC marker present in all injected cells and some host RGC). Immunohistochemistry shows cell bodies expressing human marker extending processes inside the retina towards the optic nerve, suggesting engraftment, for all samples injected in hydrogels. No such findings were seen cells injected in PBS. **b**. Statistical analysis, using one-way ANOVA followed by Tukey’s test, of the percentage of engrafted hRGC per layer after 1-month. *N* = 60 field of views were analyzed to localize injected cells in their retinal layer and extrapolate the percentage of engrafted cells. hRGC engraftment percentage was significantly higher in hydrogels groups compared to PBS (****p < 0.0001) in the target layer (RGC): IPN50 showing a higher engraftment (high average showed with center line) and narrower distribution (smaller box corresponding to 75% interval and smaller whiskers corresponding to SEM) compared to other hydrogels (*p = 0.01). Cell size and orientation were measured for more than 1×10^3^ cells and is shown as a polar plot for all groups in **c** Cells were divided into three categories: undifferentiated round cells (*r* < 30 um), medium-size processes extension (30 < *r* < 150 um) and long processes extension (*r* > 150 um). All long cells were observed only in the hydrogel groups, being parallel to the retina. **d** Colocalization analysis of cells extended processes. All events were located in the co-localization region, confirming the presence of both STEM121 and Brn3b markers in injected cells.

The core result of our work is the in vivo experimental demonstration that IPN made of Gtn-HPA and HA-Tyr enhance engraftment and extension of processes from encapsulated hRGC. We injected 5 × 10^4^ hRGC incorporated in 3 ul of Gtn-HPA, IPN75, IPN50 or PBS into the vitreous of immunosuppressed Long Evans rats. A 1-month study was performed to measure the impact of the gels on cell engraftment, using STEM121 (human marker; green) and the intrinsic Brn3b-TdTomato (RGC marker present in all injected cells and some host RGC; red) expression. Tiling images of the entire retina (Supplementary Fig. 12) show successful injections with the presence of injected cells 1 month after injection in the center of the retina. A major difference was observed (Fig. 4a) in cell morphology among groups. Cells which had been delivered in gels were found to have processes extended toward the optic nerve (long axons). Transplanted RGCs can engraft and penetrate the retina since during their migration through the gel (i.e., exiting the gel) to enter the ILM, the gel degrades through the release of enzymes from RGCs and enzymes in the vitreous^33^.

To quantify the result of our in vivo xenotransplantation we created an image processing algorithm (Supplementary Figure 13) that enables for the localization (retinal layer), size, and orientation of a substantial number of injected cells (*n* = 60 fields of view). Most engrafted injected cells (40%), for all groups, were found in the RGC layer (Fig. 4b) while the rest were mainly in the inner nuclear layer (Supplementary Data). No significant number of cells was found in the outer nuclear layer or in the vitreous (<4%). Of the initial 5 × 10^4^ injected cells, the following are the percentages of the viable engrafted cells found 1 month after injection: 52% for Gtn-HPA; 53% for IPN75; 56% for IPN50; and 38% for PBS. A noteworthy finding was that in the target RGC layer of the retina, there was a 2-fold greater number of cells delivered by the IPN50 gel compared to the saline injection group (Fig. 4b). Moreover, the IPN50 group displayed the narrowest distribution of cells in the target layer.

The ability of injected cells to extend processes is the first critical aspect to induce retinal regeneration and possible functional recovery in diseases affecting the retinal ganglion cells^34^. To demonstrate the cell engraftment enhancement offered by the IPN gels we analyzed injected cells, found in the target RGC layer, for their cell body size (including processes) and their orientation in the retina: native RGC extending parallel to the retina cross-section^35^. Figure 4c presents, in polar coordinates, the size of these cells (r) in the function of their relative orientation to the retina (θ). Most elongated cells were found in the IPN50 and Gtn-HPA groups and have their orientation close to 0 degrees, while none were found in the PBS group. The distribution of medium-size cells is centered around 0 degrees for all groups with significantly more medium-size cells found in IPN50 compared to all other groups. Finally, the number of round and undifferentiated cells was uniformly distributed through size and orientation for all groups. To corroborate that the large extensions are indeed injected cells, and not artifacts, we used a co-localization algorithm^36^, which measures the correlation between STEM121 (staining for human cells) and the intrinsic Brn3b-TdTomato (marker for injected cells and some host RGC) expression (see Supplementary Fig. 13 for more information). All events were found to have a co-localization correlation coefficient higher than 70% which corresponds to the grey area in Fig. 4d.

Immune response and Muller cell activation assay were also performed by checking and analyzing the surface coverage of cells with immune markers (IBA1 and CD45), activated Muller cell marker-GFAP (Supplementary Fig. 14), and activated microglia (CD11b and CD68) for all groups (Supplementary Fig. 15). The immune cell response was found to be significantly higher for cells injected in PBS while the immune response to cells delivered by the gels was similar to SHAM. A similar trend was observed for GFAP expression suggesting a higher retinal disturbance with PBS injections.

## Discussion

Prior studies addressed the advantages of Gtn-HPA as a carrier for subretinal injection of photoreceptor progenitor cells^37^: enzyme-mediated covalent crosslinking; independently tunable gelation rate and cross-link density; protection of cells from shear stress imparted by injection through a small-bore needle; and immune-isolation to-protect incorporated xenogeneic cells against immune cell attack. The current work demonstrates the benefits of admixing HA-Tyr with Gtn-HPA for intravitreal injection of hRGC to potentially induce functional recovery and regenerate vision lost during diseases such as Glaucoma: extending the range of the moduli of the gel; improving the attachment to the ILM; enhancement of engraftment of hRGC to the target layer; and facilitating the adoption of a natural RGC morphology of delivered cells^38^.

The use of HRP-mediated crosslinking hydrogel draws the possibility of long-term postinjection residual activity. However, no adverse cellular response to the residual activity of HRP has ever been reported in the literature reports of in vivo injection of the gel^22,39,40^. Moreover, in vitro studies of the effects of HRP concentration on cell viability have been reported^41^. The amount of HRP that was used in the gel is below that which was shown to affect cell viability.

The average pore size of HRP-mediated gelatin gels is around 100 nm. This pore size is substantially smaller than the size of a cell. However, cells are able to migrate through the gel because they release MMPs that degrade the gel to open channels for their migration^42^. Another study has shown that the initial pore diameter of the gel does not affect cell viability and the ability of the cells to migrate^7^.

Vitreous is one of several tissues in the body—including the hyaline cartilages—that comprises an IPN of collagen (principally type II) and HA. The IPN structure of the collagen and HA in these tissues imparts special mechanical behavior (*viz*., modulus of elasticity) which, in the case of the vitreous, is critical to support the surrounding ocular structures including the retina. A unique feature of the collagen-HA network in these tissues, enabled by the IPN structure, is the dynamic control of modulus offered by the HA uptake of water: the swelling of the vitreous, which is due to a high fixed charge density. Therefore, stiffening the tissue by extending the collagen fibrils of the network^43^.

The formation of an IPN from the mixing of Gtn-HPA and HA-Tyr was unexpected as both side groups can theoretically chemically react and bind to each other, which was not observed in our experiment. It is still unclear why the mixing of Gtn-HPA and HA-Tyr formed an IPN, as opposed to a random copolymer network, as both side groups can theoretically chemically react and bind to each other, which was not observed in our experiment. While collagen-HA IPN have previously been prepared for several applications using various methods^44,45^, none have offered the degree of in vivo tunable gelation rate and cross-link density for an injectable gel^46,47^, as provide by the gelatin-HA formulation described in the current work.

In a prior study in which 5 × 10^4^ labeled rat RGC suspended in 2 μl of PBS were injected intravitreally into adult rats^48^, cells from a wide range of donor ages, but not from adults, were only occasionally found to have migrated into the ganglion cell layer. In other work, in which 40,000 or 60,000 rat RGCs in 3–4 ml of serum-free medium were injected intravitreally in rats, the exogenous cells migrated into, and established themselves in, the ganglion cell layer. They appeared to “extend axons toward the optic nerve head of the host retina and dendrites growing into the inner plexiform layer.”^49^ Electrophysiological recordings demonstrated the electrical excitability and light responses of the transplanted cells.

The comparison of volumes injected in the rats could be used to derive the rough estimate of the number of cells that would be needed in clinical trials or in higher animals like rhesus monkey. Rats possess around 50,000 RGCs in a single layer in their entire retina while big animals and human possess around 1 M (with more than 50% located around the fovea^50^). Using the calculation, a volume of 100 uL with 500,000 cells should be used for big animals in a clinical setting to replace the dead RGCs in the central retina.

Recent findings have demonstrated the ability of the ILM and the ECM to signal and limit the engraftment of injected RGCs. These interactions are also promoted by the degradation of enzymes (such as MMPs) to support or prevent the engraftment postinjection^51^. There are many other molecular signals, such as CAM^52^, Muller glial end feet^53^, or netrin^54^ which can be studied due to their presence in the adult retina and their impact on RGC integration.

Our supposition is that incorporation and delivery of RGCs into the gelatin-HA gel primes them for their migration to and establishment and function in the retinal ganglion layer of the host, by exposing them to the correct chemical (such as thrombospondin^55^) and mechanical cues that effect their expression of cell adhesion molecules and other receptors, including those that control extension of afferent and efferent cell processes.

To create a system that can break through the ILM without presenting hostile environments to the encapsulated cells, it was critical that the animal model used, did not have existing additional challenges. While the RGCs anatomical connection were achieved by using the IPN as a delivery vehicle, the critical remaining proof of retinal regeneration is still needed: functional and behavior recovery. The limitation of this work was that functionality could not be assessed since Long Evans rats do not have the optic nerve or RGC injury. However, the low immune response, along with cell attachment, suggest a possible enhanced functional recovery which needs to be proven in an adequate model. In the future improvement of this work, we plan to evaluate the effect of IPN, encapsulating RGCs, in a glaucoma animal model and identify the efficacy of this system and its ability to restore the function of the degenerating retina by performing functional recovery tests such as ERG, OCT, and VEP.

Overall, we expect this work to provide design principles for drug and stem cell therapies in the eye and other organs which depend critically on the viability and stability of cells during injection and development.

## Methods

### Hydrogels’ preparation

Gtn-HPA and HA-Tyr conjugate were prepared via a general carbodiimide/active ester-mediated coupling reaction (in PBS) that conjugated HPA to gelatin (MW 20-80kDA) and Tyramine to Hyaluronidase (MW 80–150), as previously described^22,56^. 90% of the amine groups were conjugated with HPA or Tyramine. For the control hydrogels, 0.1 U/mL of horseradish peroxidase HRP (Wako USA), and 1 mM of H2O2 (Sigma-Aldrich) with or without hRGC, were mixed into Gtn-HPA or HA-Tyr hydrogel prepared using 2 wt% solution of Gtn-HPA or 0.25, 0.5 and 2%wt solution of HA-Tyr. Other concentrations of crosslinker H2O2 (0.5, 0.8, 0.9, 1, 1.3, 2.5, and 5 mM) were used to investigate the effect of hydrogels on cell viability and shear modulus (which was measured with oscillatory rheology).

Hybrid interpenetrating networks (IPN10, 25, 50, 75, and IPN90) were prepared by mixing the corresponding amounts of Gtn-HPA and HA-Tyr at 2 wt% solution (e.g., IPN75 corresponds to 75% of Gtn-HPA and 25% of HA-Tyr both at 2 wt% solution). To create IPN hydrogels, 0.1 U/mL of HRP and different concentrations of H2O2 (0.8, 0.9, 1, and 1.3 mM) with or without cells were mixed into the solution. Hydrogels were formed after less than 5 min and incubated at 37 C to reach stability^7^.

Hyaluronic acid with high molecular weight (HHA) (MW 1200 kDA, Sigma-Aldrich) was dissolved in PBS at 2, 3, and 5%wt solution with hRGC by thoroughly mixing the samples with a vortex throughout the preparation. No chemical reagent was added and physical crosslinking (chain entanglement) was seen in less than a minute.

Collagen-Genipin (CG) hydrogels were prepared by dissolving soluble collagen type I from calfskin (Sigma-Aldrich) in a 2%wt solution and mixing it with hRGC. Genipin 98%-HPCL (Sigma-Aldrich) at concentrations of 0.5, 1, 5, and 10 mM were then added to the solution containing polymer and cells. Hydrogels were incubated at 37 C and reached stability after 20 min^57^.

### Oscillatory rheology

Oscillatory rheology was performed with a TA instruments AR-G2 rheometer using cone and plate geometry of 40 mm diameter and 2° angle. For each measurement, 200 μl of each sample (Gtn-HPA, IPN90, IPN75, IPN50, IPN25, IPN10, and HA-Tyr) at 2%wt/vol, containing 0.1 U/ml of HRP and varying concentrations of H2O2 (ranging from 0.8 to 1.3 mM) was applied to the bottom plate immediately after mixing. All hydrogels having a gelation time comprised between 30s and 3 min samples were still liquid when applied onto the bottom plate. The upper cone was lowered to a measurement gap of 51 μm. As soon as a layer of silicone oil was applied, to prevent evaporation, the rheometer was started. All measurements were taken at 37 °C in the oscillation mode with a constant strain of 1% and frequency of 1 Hz. To estimate the gelation rate, the time at which the gel point (as defined by the crossover between storage modulus, G′ and loss modulus, G″) occurred was measured. G’ (storage modulus) and G″ (loss modulus) were measured every 2 s. The final plateau value of G’ and time to reach this plateau were then recorded for each sample. Due to the fast gelation of all samples and time to stick the sample onto the bottom plate and the start of the experiment, the gel point was not measured with oscillatory rheology. Measurement can be seen in microrheology experiments.

### Differential scanning calorimetry (DSC) and Gel permeation chromatography (GPC)

All materials were analyzed using a differential scanning calorimeter (DSC 250) (TA Instruments, New Castle, USA). Polymer powders (Gtn-HPA and HA-Tyr) were analyzed from 20 to 250 C at a heating speed of 10 °C/min. Glass transition was observed by measuring the derivative of heat flow and melting temperature was seen as a peak above the glass temperature.

Gel permeation chromatography was performed at 35 °C with a Malvern Viscotek VE 2001 GPC max UV 2501 detector, a TDA 301 chromatography system, and a PL aquagel-OH MIXED-M column (Agilent). Samples were run at 1 mg/mL through a mobile phase comprised of 10 mM sodium phosphate monobasic (Macron Chemicals), 100 mM sodium nitrate (Sigma–Aldrich), 20%wt/mL methanol (Sigma–Aldrich), adjusted to pH 7.4. HA-Tyr and Gtn-HPA were dissolved in 2 mL of the mobile phase, at a concentration of 10%wt/mL, by thoroughly mixing and incubating samples for 1 h at 37 C. Molecular weights were then referenced against polyethylene glycol standards (Waters). Molecular weight parameters (Mw, Mn, P, and MWD) were calculated for standards and samples using the respective GPC calibration equation: Log(Mn) = Ao +A1*Vp, where Mn is the molecular weight, Vp is the eluded volume, Ao=10.2086 and A1 = −0.7604. Chromatogram heights were measured at retention times of interval 0.5 min.

### Compression test study

Unconfined compression tests were performed using a Zwick/Roell Z2.5 static materials tester (Zwick GmbH & Co., Ulm, Germany) with integrated testing software (testXpert, Zwick). 1 mL of Gtn-HPA, IPN50, IPN75, and HA-Tyr were prepared into 24-well plates to create samples 16 mm in diameter and 3–4 mm in thickness. All hydrogels were left to fully crosslink and stabilize for 2 h at 37 C before performing compression testing. All gels were swelled in PBS for 1 h before compression testing.

Mechanical tests were performed at a constant strain rate of 0.5%/s to a maximum strain of 10% using a 20 N load cell (Part No. BTC-LC0020N.P01, Zwick) sampling at a frequency of 2 Hz. The diameter of the samples at the start of the testing was measured using digital calipers. The compressive modulus was determined by the slope of the true stress-strain curve within the linear regime of the material (~0-7%).

### Fourier transform infrared (FTIR) spectroscopy analysis

Fourier transform infrared (FTIR) spectrum was recorded to detect the chemical and structural nature of Gtn-HPA powder, HA-Tyr powder, and dried hydrogels (Gtn-HPA, IPN75, IPN50, HA-Tyr), using a Thermo Fisher FTIR6700 spectrometer. Samples were characterized using attenuated total reflection (ATR) mode for a total of 32 scans in the range of 500–4000 cm^−1^. FTIR baseline was applied and normalization was performed with respect to the characteristic backbone peak (around 1600 cm^−1^ for Gtn-HPA and 1000 cm^−1^ for HA-Tyr). For dried hydrogels spectra, 10 mL of each sample was prepared at a concentration of 10 wt%/mL, casted into a 5 cm petri dish. Samples were left to dry overnight in a low oxygen incubator (37 C, 5% O_2_, and 5% CO_2_).

### In vitro degradation assays for hydrogels

200 μl gels (Gtn-HPA, IPN75, IPN50, IPN25, and HA-Tyr) were prepared as previously described in section Hydrogels preparation and incubated for 30 min at 37 C to reach stability. Samples were then combined with 200 ul of phosphate-buffered saline (PBS) containing 1000 U/ml type IV collagenase (Invitrogen) or containing 500 U/mL hyaluronidase type I-S (Sigma-Aldrich) and incubated at 37 °C on an orbital shaker at 150 rpm. Samples were collected every 5 or 10 min for 1 or 2 h, for collagenase or hyaluronidase treatments respectively, and analyzed for degradation products using the bicinchoninic assay (Thermo Fisher Scientific).

In order to model and replicate the in vivo conditions, slow degradation assays were also performed. 200 ul of the injected hydrogels (Gtn-HPA, IPN75, and IPN50) were prepared and incubated for 1 h at 37 C to reach stability. As suggested and explained in^30,31^, the actual concentration of collagenase (coming from MMPs) and hyaluronidase (intrinsic in the vitreous) are respectively 0.5 U/ml and 0.3 U/ml in vivo.Five ml of the enzymes with these concentrations were used, mixed together, and added to the hydrogel samples. Samples were kept in incubators, collected every day for 9 days, and analyzed for degradation products using the bicinchoninic assay. Degradation rate constants were derived by fitting the data for mass loss into an inverse exponential model.

### Passive microrheology measurements and PLGA microbeads tracking

All samples having an extremely short gelation time, common oscillatory rheology was not able to capture their gel point. Hence, to characterize this specific viscoelastic characteristic passive microrheology was performed. A volume of 10 ul of 10–20 um PLGA microbeads (Sigma-Aldrich), at a concentration of 10^5^/mL, were thoroughly added and mixed with all polymers. Then, after the addition of the catalyst (HRP) and the crosslinker (H_2_O_2_), 200 ul of each sample was pipetted as fast as possible onto a microscope slide and particles movement were tracked for a period of approximately 4 min by taking a video of their Brownian motion inside the hydrogel in process of gelation.

ImageJ (Fiji, NIH) was used to track the center of *n* = 15–17 particles for each sample during 6 min. A MATLAB program was then used to track the particles, calculate the instantaneous mean square displacement (MSD) and fit the data with a double exponential decay as seen in Fig. 1d. The MSD of *n* = 15 particles was calculated and averaged every 2s^58^. This fit function was then used to calculate the complex modulus by feeding the data into a MATLAB function that fits this data with a second-order polynomial function from which the first- and second-time derivatives are computed and from that the complex modulus^59^. Finally, the storage modulus (elastic) G′ and loss modulus (viscous) G″ were measured in order to calculate the gel point (defined by the crossing of G′ and G″).

### Source and viability of hRGC

Human retinal ganglion cells (hRGC), gifted from Dr. Donald Zack laboratory, were described in previous studies^28,60^, being Brn3b-TdTomato positive due to the isolation and labelling process. Cells at 1 ×10^5^/mL, suspended in saline or medium (mTeSR1 media: Stemcell Technologies) were added to 1 mL of the IPN formulations, along with different catalyst and crosslinker concentrations, listed in section Hydrogel preparations, and were pipetted onto fibronectin-coated round glass coverslips (thickness 5 mm, diameter 1 cm, VWR). After 1,3,5 and 7 days of incubation with medium or PBS, they were incubated with 2.5 µM calcein-AM (FITC) and 10 µM ethidium homodimer-1 (Cy3) in PBS for 15 min at 37 °C and 5% CO_2_. hRGC were then washed three times with PBS for 10 min, at room temperature. Coverslips with cells encapsulated in hydrogels were mounted on poly-l-lysine microscope slides (thickness 1 mm, L x W 75 × 25 mm, Thermo Scientific Shandon) with low viscosity slide mounting medium (Fisher Scientific) before imaging with an epifluorescence confocal microscope (Leica SP8, USA), in order to capture the 3D configuration of cell distribution through the different hydrogels.

### Harvesting hRGC for Flow cytometry

hRGC [(3 ×10^5^ cells/mL in PBS or in 1 mL of Gtn-HPA, IPN75, and IPN50 hydrogels, in 6-well plates (3.5 cm diameter, polystyrene, flat bottom, sterile, fisher scientific)] were maintained in PBS for 5 days (replicating the in vivo conditions). 1000 U/mL collagenase-type IV (Invitrogen) was then added to degrade the Gtn-HPA hydrogels and 500 U/mL hyaluronidase type I-S (Sigma-Aldrich) to degrade the HA-Tyr part of the IPN; after 20 min of incubation, Gtn-HPA, IPN75 and IPN50 gels were fully dissolved. Samples were centrifuged, and hRGC were harvested. The phenotype of hRGC was analyzed using flow cytometry with the MACSQuant flow cytometer (Miltenyi, San Diego). hRGC, from the 4 different conditions—in PBS, in Gtn-HPA, IPN75 and IPN50 were collected and fixed with a Perm/Fix buffer (BD Biosciences) at 4 °C for 15 min. Cells were then washed in a wash buffer (BD Biosciences) and incubated, at room temperature, in a blocking buffer (Pharmingen staining buffer with 2% goat serum) for 30 min. Blocked cells were seeded onto a flat-bottom 96-well plate (treated, sterile, polystyrene, Thomas Scientific) and labeled overnight at room temperature with the following conjugated primary antibodies: Brn3a-FITC, RBPMS-APC, Thy1.1-APC (ganglion cell marker), Caspase9-FITC (apoptosis marker), Ki67-FITC (proliferation marker), Cmyc-FITC, Oct4-APC (stemness markers) and NeuN-APC (neuronal marker). Primary antibodies were diluted in 200 uL of antibody buffer (TBS, 0.3% Triton X-100 and 1% goat serum). After overnight incubation cells were washed three times for 15 min, and incubated in secondary antibodies and left at room temperature for 3 h; secondary antibodies (goat-derived anti-rabbit and anti-mouse, DAPI-VioBlue) were diluted 1:200 in antibody buffer (Jackson Immunoresearch Laboratory). Light scatter and fluorescence signals from each sample were measured using the MACSQuant (Miltenyi Biotech, Germany) flow cytometer (2 ×10^5^ events were recorded). The results were analyzed using the MACSQuantify software (https://www.miltenyibiotec.com). For each primary antibody the DAPI-positive single cell population was gated. The ratio of positive cells in the gated population was estimated in comparison with blank and species-specific isotype controls. Primary antibodies and their dilutions are listed in Supplementary Table 1.

### In vivo xenograft study—animals, surgery and tissue processing

The research protocol was reviewed and approved by the Schepens Eye Research Institute Animal Facility and was in accordance with the Association for Research in Vision Ophthalmology Statement for the Use of Animals in Ophthalmic and Vision Research. Twenty-seven female Long Evans rats (age 12 weeks, approximate weight 200 g, Charles River. Wilmington, MA) were used in the experiments. Transplantations were performed on Cyclosporine (Atopica, oral solution 100 mg/mL, Novartis, USA) immunosuppressed rats. Animals were sedated using 2–3% isoflurane (Abbott, Solna, Sweden, http://abbott.com) in combination with oxygen by placing the rats in an inhalation chamber, followed by intraperitoneal injection of ketamine (40–80 mg/kg) and xylazine (10 mg/kg) for anesthesia. Eyes were first anesthetized using topical ophthalmic proparacacome (0.5%) followed by Genteal to keep the lens moist during the surgery.

A conjunctival incision and a small sclerotomy were performed using a fine disposal scalpel in all rats. A 2%wt Gtn-HPA/hRGC hydrogel (*n* = 5), 2%wt IPN75/hRGC (*n* = 5), 2%wt IPN50/hRGC (*n* = 5), or a cell suspension in PBS (*n* = 5), were injected into the vitreous of the rats. 3 rats were taken as control and n = 4 rats were subjected to only SHAM injection. All injections were performed using a glass pipette (internal diameter, 150 µm) attached to a 10 uL Hamilton syringe via a polyethylene tubing. Approximately 5 ×10^4^ cells in an injection volume of 3 µL were used in each of the 4 groups. The presence of islands of gels onto the back of the eye was checked using a glass coverslip applied to the eye. The vitreal injection was considered successful if shiny islands were seen under the dissection surgical microscope (Alcon Vitreoretinal, Constellation Vision System). Triple antibiotic (Bac/Neo/Poly) was given locally at the end of the surgery to prevent infection. The rats were then placed in their cages for 4 weeks. 100 mg/L of Cyclosporine was added to the water container of all cages and was changed every 3 days.

Four weeks post transplantation, immunosuppressed rats were sacrificed by CO_2_ inhalation for 5 min. Cervical dislocation was performed to certify death. Eyes were enucleated and placed in 4% paraformaldehyde for 24 h. Tissues were subsequently saturated with increased concentrations of sucrose (5%, 10%, 20%) containing Sorensen phosphate buffer. Eyes were immersed in 30% sucrose overnight or until dissection. The tissues were embedded in cryosection gelatin medium overnight and sectioned at 15 µm thickness on a cryostat. During the sectioning process, every 4th section was stained and examined by epifluorescence for hRGC presenting with STEM121-FITC (human cell marker) and DAPI-VioBlue (cell nucleus). Every 5^th^ section was stained with CD45-PE, IBA1-FITC (immune cell marker) and DAPI-VioBlue. Every 6th section was stained with GFAP-PE (Muller cells marker) and DAPI-VioBlue.

### Vitreal injections and optical coherence tomography imaging (SD-OCT)

The same protocol for sedation and anesthesia as for the xenograft study was used for vitreal injections of hydrogels and SD-OCT imaging. Rats left pupils were dilated with tropicamide (VetRXDirect, USA). Animals were then anesthetized and a conjunctival incision and a small sclerotomy were performed using a fine disposal scalpel in all rats. A 2%wt Gtn-HPA hydrogel (*n* = 5), 2%wt IPN75 (*n* = 5) and 2%wt IPN50 (*n* = 5), all samples without cells, were injected into the vitreous of the rats. Animals were then placed in front of the SD-OCT imaging device (Spectralis HRA + OCT, Heidelberg Engineering, MA, USA). Eyes were kept moisturized with HBSS during the whole procedure. Images were taken before, right after injections, 1 h after and each day until no more gel was visible. The presence of gel was assessed by visible islands of gel sitting on top of the retina in IR images. Images of the back of the eye with 4B-scans 30 frames were taken and retinal sections with 4B-scans 60 frames, all done in the rectangular scan. Acquired images were saved as.tiff files. First, image artifacts due to breathing movements were eliminated by using the StackReg Plugin. Then, all frames were converted into a single image by applying the z-projection. This average enables for the elimination of most of the noise observed on individual images, which help to see the presence of gel and its volume, or degradation time. Comparison before and after injection was performed to see the impact of gel injection on retina morphology and detachment.

Three days post vitreal injection, rats were sacrificed by CO_2_ inhalation for 5 min. The cervical dislocation was performed to certify death. The same protocol as for xenograft study was applied to enucleated eyes. After sectioning, every other section was stained and analyzed with Hematoxylin and Eosin (H&E) in order to measure and locate the different gels on top of the retina.

### Immunofluorescence and histological staining of in vitro samples and in vivo cryosections

hRGC cultured on microscope coverslips, and cryosections from Long Evans rats left eyes, were fixed with 4% paraformaldehyde in 0.1 M PBS (Irvine Scientific) at room temperature for 20 min. These fixed cells and sections were blocked and permeabilized with a blocking solution [(Tris-buffered saline (TBS), 0.3% Triton X-100 and 3% goat serum (Jackson Immunoresearch Laboratories, West Grove, PA, http://www.jacksonimmuno.com)] for 15 min. Samples were then rinsed twice with 0.1 M TBS buffer for 15 min each time, mounted on polysine microscope slides and incubated with primary antibodies overnight at 4 °C (Table 2): Brn3a-FITC, RBPMS-FITC, Thy1.1-FITC (ganglion cell marker), Caspase9-FITC (apoptosis marker), Ki67-APC (proliferation marker), Cmyc-APC, Oct4-APC (stemness markers) and NeuN-APC (neuronal marker), CD11b-FITC and CD68-APC (Microglia maker), CD45-FITC (leukocyte marker), IBA1-FITC (macrophage marker), GFAP-FITC (muller cell marker) and STEM121-FITC (human cytoplasmic marker with no cross-reactivity with rats). After overnight incubation, samples were rinsed three times with TBS for 15 min. Secondary antibodies (goat-derived anti-mouse and anti-rabbit, DAPI-VioBlue) staining was performed for 1 h at room temperature. Samples were then washed a final time with TBS before being mounted on poly-l-lysine microscope slides with low viscosity slide mounting medium. Digital images were obtained with an epifluorescence confocal microscope (Leica SP8) using 63x-oil objective.

**Table 2:** Name, isotype, working dilution and source of antibodies used for flow cytometry and immunohistochemistry.

Antibody	Isotype	Working Dilution	Source
**DAPI-VioBlue**	IgG2a	1:1000	BD Biosciences
**Caspase9-FITC**	IgG1	1:50	Santa Cruz
**CMYC-FITC**	IgG1	1:200	BD Biosciences
**STEM121-FITC**	IgG1	1:50	TakaraBio
**Brn3a-FITC**	IgG1	1:100	ThermoFisher
**IBA1-FITC**	IgG1	1:100	Abcam
**KI67-APC**	IgG2a	1:50	Santa Cruz
**RBPMS-APC**	IgG2a	1:100	EMD Millipore
**THY1.1-APC**	IgG2a	1:100	Miltenyi Biotech
**NeuN-APC**	IgG2a	1:100	Abcam
**Oct4-APC**	IgG2a	1:200	BD Biosciences
**CD45-PE**	IgG H&L	1:100	Abcam
**GFAP-PE**	IgG H&L	1:50	TakaraBio
**Rb Control**	IgG1	1:100	Abcam
**Ms Control**	IgG2a	1:100	Abcam

Slides from the vitreal injection of gel only were examined using H&E staining, 3 days postinjection. Thermo-scientific Rapid-Chrome H&E staining kit was used. This consists in an 18-steps process, which permanently stains cytology specimens. Slides are dipped into a series of solutions containing 95% alcohol, distilled water, Hematoxylin, Bluing reagent, and Eosin-Y stain followed by a series of washings before the final fixing step. Slides were then mounted and observed under an upright microscope (Leica DM2500) at different magnifications.

### Confocal microscopy and cell analysis via image processing

All stained samples (except H&E staining) were analyzed, and images were taken using Leica SP8 confocal microscope. Images were taken with sequential scanning at 1024 × 1024 or 2042 × 2042 resolution with the following lasers intensity and characteristics: VioBlue-PMT at 5.4% with line average of 3 and gain of 875 V, FITC-HyD at 2.3% with line average of 3 and gain of 77%, PE-HyD or APC-HyD at 3.7% with line average of 3 and gain of 85%.

hRGC viability images were taken at 20x magnification with a z-stack of 300 um and 22 steps. A 3D projection was used for qualitative analysis while maximum projection was applied as quantification. Cells in 50 randomly selected maximum-projected fields of view were counted under 20x objective lens magnification with a cell counting and analyzing image processing algorithm^61^. This ROIs selection enables for more than the quantification of more than 75% of each sample, reducing the possibility of ROIs selectivity.

To apply this algorithm, we converted each image to a greyscale (from the specific staining analyzed live: green and red: dead). To be able to analyze cells correctly, multiple image rendering processes were used. The extraction of dimmer cells was performed by contrast adjustments. The elimination of objects on the borders (which can cause noise and be artifacts) was realized with an intrinsic MATLAB function. Noise removal, critical to extract only cells and not artifacts, was done with adaptive filtering (small window). The final image rendering included using a global Otsu’s thresholding method to convert the image to binary, filling the image region and holes, performing a morphological opening using a disc kernel and finally removing all small cells (connected components with the low number of pixels <10px). The final image can be seen in Supplementary Figure 6b. To further analyze cells, we performed perimeters extraction of cell or cell groups, as seen in Supplementary Figure 6c. Some cells might be grouped and counting their number may be critical for viability results. To extract cells in groups we applied a watershed algorithm, which can divide the groups into distinct cells. The watershed algorithm interprets different levels of gray intensity, in an image, as altitude. It then finds objects which are delimited by their perimeter with a high altitude in their center (high overlapping intensity). To implement the watershed algorithm, we modified the image by finding the maxima (corresponding to the cell nuclei) and transformed the image to show the perimeter and these maxima (Supplementary Figure 6d). We finally applied the watershed algorithm which finds all connected components and enables for an easy cell counting (Supplementary Fig. 6e). Cells number (green for live and red for dead), size (area of positive pixels) and shape factor (which corresponds to the relative shape of an object compared to a circle: C = (4 * π * A)/(P^2^), C = 1 being a perfect circle) were measured for each field of view. A cell with a low shape factor could be due to elongated or stellate morphology resulting from the formation of cell processes. Due to small cell size, while most cells have been correctly detected, some have been taken as dust and deleted.

After 1, 3, 5, and 7 days of culture, the percentage of viable cells was calculated by dividing the number of live cells (FITC) by the total number of cells in the given area (live and dead cells added). The same z-stacks images were used to measure the distribution of the cells through the hydrogels. As seen in Fig. 2c, LASX Leica software enables to measure the average intensity of a marker along each (x,y,z) direction. These intensities were measured for each z-stack, in each group after normalization, and used averaged.

Qualitative immunofluorescence staining (RGC, stemness, neuronal, proliferation, and apoptosis markers) along with immune and Muller cell activation markers were taken at 20x of 63x with oil objective magnification with a z-stack of 15 um and 25 steps. Images, after maximum projection, in 15 randomly selected fields of view were used to quantify the surface coverage of specific markers (IBA1, GFAP and CD45). To do so a MATLAB algorithm was created that consisted in calculating and counting (with a tolerance of 0.01%) the number of colored pixels (Green for FITC channel and Red for PE or APC channels) for each marker. Then, a percentage compared to the total size of the image was created which corresponded to the surface coverage of the specific marker.

Entire tiling of the retina was performed on all injected groups (Gtn-HPA, IPN75, IPN50 and PBS) at 63x with oil objective magnification and z-stack of 20 um with 50 steps. The tiling square size was 25 × 10 fields of views, which was reduced by only choosing the field of views containing parts of the retina as seen in Supplementary Figure 12.

Finally, to be able to quantify the improvement of injected cells migration and engraftment into the retina of rats, STEM121-FITC with DAPI-VioBlue and Brn3b-TdTomato staining were analyzed in a larger quantity than all other images. Images were taken at 63x magnification with a 15-um z-stack and 22 steps. For each group, 60 fields of view, chosen in the center of the retina (where the injection was performed) were analyzed.

### Hydrogels-ILM interface and OCT analysis algorithms

Each slide, stained with H&E, was analyzed under bright-field light microscopy at 20x magnification to find the location and size of the different hydrogels after three days. To quantify this measurement, 10 fields of view were used to look at the interface of retina and vitreous. Interfaces were analyzed as follow were gel was visible (attached or not to the retina) the interface was split into 200 um parts (usually 2-3 per image); the interface of each part was then characterized by looking at the region of attachment (red regions in Supplementary Figure 10) and the others (white regions in Supplementary Figure 10). Afterwards, the percent of attachment for each interface (proportion of red regions compared to white regions) and the distribution of the gel at the interface (holes in the red regions) were calculated using a MATLAB algorithm. This enables for the characterization and measurement of the attachment of the different gels to the retina after three days.

Islands of gels were visible during SD-OCT data acquisition and a quantification of gel presence was performed by applying an image processing thresholding algorithm. This algorithm was based on Otsu’s method of thresholding^62^ and enables a specific quantification of the gel degradation in vivo. After taking the OCT images of the section of the retina, 10 fields of views were analyzed for each group. In order to apply and use Otsu’s method, the retina and the noise were processed to be considered as the background while the gels were processed to be considered as the foreground. Most of the noise was deleted by the plugin in the SD-OCT software, hence the rest of the image was just composed of a bright retina (curved with different layers) and islands of gel on top of it. In order to process the retina as background, different values of the presence of the retina with no injections were calculated: this consists in counting the number of foreground pixels found with Otsu’s algorithm in OCT images without injection. These values were used as a normalization in order to find the surplus of foreground pixels where gel was present. This quantification was performed for all groups at all time points and an in vivo degradation curve (which was confirmed with in vitro degradation) was calculated (Fig. 3c).

### Image processing algorithms for detection of cell migration, co-localization, and orientation compared to retinal layers

The improvement in engraftment of encapsulated hRGC was quantified by measuring cell migration (location in retinal layers after 1 month), co-localization (expressing both intrinsic Brn3b and STEM121 human markers) and, for large cells, orientation (angle formed by extended processes and retina). After using Leica SP8 confocal microscope to image the test groups (60 fields of view per group), as explained earlier, colored images (VioBlue for DAPI, FITC for STEM121, and PE for TdTomato) were analyzed.

To segment cells from images, we designed a two-step algorithm (Supplementary Data). We first normalized pixel intensities of input images from 0 to 1. Due to the large amount of noise in the image, we started the segmentation pipeline with a small amount of gaussian blur to smooth the image. Part of the cells were very dim, therefore, to segment them, we used a 1^st^ low threshold (around 10% of intensity) to segment cells. To remediate cells which displayed small holes, we incorporated a closing morphological operation (consisting of a dilation followed by an erosion). We also ran an opening (an erosion followed by a dilation) to remove small clusters of pixels that were most likely noise. For large cells, the low threshold followed by morphological operation led to a high recall for cell pixels. We then segmented the brightest part of cells using a 2nd larger threshold (around 0.3). To merge those two segmentations, we designed a fusion algorithm. Using the rough segmentation obtained from the 1st threshold, we employed a connected component algorithm to label each independent group of segmented pixels. Then, we eliminated each labeled group of segmented pixels which did not contain pixels from the high threshold segmentation. The final result had a high precision (high threshold) and a high recall (low threshold). We used labelled groups of segmented pixels to compute the area of segmented cells. This extraction worked with most cells however, precision suffered: some pixels—especially around the border of the image—were above the threshold, leading to segmentation toward unwanted parts of the image. To solve this issue, we decided to delete the 5px border of each image (which corresponded to <1% of the image) usually just removing 1–2 cells per picture. To set a constant and relatable quantification we perform an intensity normalization for all images and were able to localize almost all cells in each image.

While analyzing the data set, we observed many cells which had started their differentiation and extension of long processes toward or in the optic nerve during their engraftment. To quantify this finding, we calculated cell size (stroma and processes) which worked well except with crescent shape cells or very round-shaped cells. Morphologically, retinal ganglion cells extend their processes towards the optic nerve parallel to all retinal layers 12, hence we also measured the relative orientation of our injected cells with the annotated layers of the retina. The actual orientation of each cell corresponds to the angle difference between its body and the layer it is located in. Both angles were measured by fitting the largest possible ellipse in both the extracted cell body and annotated layers (cell ellipses shown in the second column) and using their long axes angle.

All images were manually annotated using an online annotation tool (Makesense.ai). This enabled the localization of each layer of the retina and therefore, by fitting labelled groups, the migration of injected cells and their location in the retina. The orientation of both retinal layers and cells was measured by fitting each group with the smallest enclosing ellipse.

To make sure that STEM121 human staining and intrinsic Brn3b expression were co-localized in regions of interest (cells) we used a co-localization algorithm previously described^36^. Using MATLAB as a basis, the co-localization program analyzed the content of images taken with confocal microscopy. First, images were filtered, thresholded, and analyzed with co-localization and Pearson’s and Mader’s algorithms. Co-localization consists in finding the fraction of pixels, which possess a high intensity in both colors (green and red in our case) with linear approximation. *P* value, Pearson and Mader’s coefficient and co-localization number were then calculated and reported. This algorithm measures the presence of both STEM121 marker and human intrinsic Brn3b for each extracted cell. It finds the M1 and M2 coefficients which correspond to the amount of respectively green and red pixels in each image while measuring the correlation coefficient of these pixels.

### Statistical analysis

All experiments were performed with *n* = 10–15 (except image processing for cell location and migration which had *n* = 60 for each group). The power calculation was based on detecting a significant difference in the means between groups of 30–40% with a standard deviation of 15% and α = 0.05 and β = 0.20. Values were expressed as mean + /- standard error mean (SEM) using GraphPad software (https://www.graphpad.com/). Analysis of variance (one-way and two-way ANOVA) followed by Tukey’s and Student’s *t* test were performed for statistical analysis. Statistical significance was set at *p* < 0.01.

### Reporting summary

Further information on research design is available in the Nature Research Reporting Summary linked to this article.

## Supplementary information

Supplementary Information

Reporting Summary

Supplementary Movie 1

Supplementary Movie 2

Supplementary Movie 3

## Data Availability

The data that support the findings of this study are available from the Gilbert foundation database platform: https://nf.synapse.org/Explore/Studies/DetailsPage?studyId=syn21650484.

## Code availability

The algorithms created during this study are available from the corresponding author upon reasonable request.
