# Supplementary material for: A bioinspired gelatin-hyaluronic acid-based hybrid interpenetrating network for the enhancement of retinal ganglion cells replacement therapy
Source: NPJ Regen Med. 2021 Dec 20;6:85. doi: 10.1038/s41536-021-00195-3 (PMC8688498; doi:10.1038/s41536-021-00195-3)
Supplement: Supplementary file 1 — Supplementary Information [file 41536_2021_195_MOESM1_ESM.docx]

***Supplementary Note***

**A bioinspired gelatin-hyaluronic acid-based hybrid interpenetrating network for the enhancement of retinal ganglion cells replacement therapy.**

Pierre Colombe Dromel^1,2^, Deepti Singh^2^, Eliot Andres^2^, Molly Likes^3^, Motoichi Kurisawa^4^, Alfredo Alexander-Katz, ^1^Myron Spector^5^, and Michael Young^2*^ ^*^

^1^Massachusetts Institute of Technology, Cambridge, MA, USA

^2^Schepens Eye Research Institute of Massachusetts Eye and Ear, Harvard Medical School, Boston, MA, USA

^3^Wellesley College, Wellesley, MA, USA

^4^A*STAR Institute of Bioengineering and Nanotechnology, Singapore

^5^VA Boston Healthcare System, Brigham and Women’s Hospital, Harvard Medical School, Boston, MA, USA

# Supplementary video – Gel point

**Passive microrheology experiment and measurement (via multiple particle tracking) for Gtn-HPA, IPN75 and IPN50**. Movement of particles, due to their Brownian motion inside the pores of the hydrogels in the process of gelation, recorded and tracked with a particle tracking program for 90s (videos are presented here with x10 fast forward). For Gtn-HPA, particles were observed to quickly stop their movement while still possessing a non-zero mean square displacement at longer time points for stiffer hydrogels (IPN75 and IPN50). These videos demonstrate the mechanical and surgical tunability of our IPN due to their different kinetics of gelation.

# Supplementary Note 1 – Characterization of Materials

## Chemical structures and broad viability testing

Chemical structures of all materials (polymers and hydrogels) used in the first biocompatibility assay, to find potential candidates for the enhancement of intravitreal injection are shown in Supplementary Figure 1. The proposed interpenetrating network formed by the mixture of Gtn-HPA and HA-Tyr can be seen in Supplementary Figure 1d.

To determine the degree to which cell viability was maintained through the covalent crosslinking process, human retinal progenitor cells (hRGC) were incorporated into the candidate gel formulations without media and cultured for 2 days. Supplementary Figure 1e summarizes this viability data (mean+/- standard error of the mean) with our negative monolayer control in PBS having only 26.5% viable cells and our positive monolayer control with medium having 91.5% viability (being significantly higher than all candidates deprived from nutrients). A threshold of 55% viability was applied to consider any sample biocompatible. Collagen-Genipin (CG) hydrogel samples (averaged for all Genipin concentrations) showed the lowest viability of incorporated cells, due to the fragility of retinal cells and the relatively high cytotoxicity of Genipin needed to produce a stable hydrogel. Due to its high molecular weight and high stiffness, HA alone showed really low viability after 2 days, even lower than PBS. By reducing the percent of HA and using the chemically induced crosslinking HA-Tyr viability reached 37-39%. By mixing HA-Tyr and Gtn-HPA at different quantities (IPN50 with 50% of each and IPN75 with 75% of Gtn-HPA and 25% of HA-Tyr), viability was higher than the desired values: reaching respectively 59% and 61%. Finally, Gtn-HPA alone showed the highest viability result, being the most biocompatible polymer, with 61.2%. There was no statistical difference measured between the three highest candidates (IPN50, IPN75 and Gtn-HPA), however they were significantly higher than all other hydrogels and PBS. The hydrogel candidates showing high biocompatibility within a first short-term viability test were Gtn-HPA and interpenetrating networks made of various content of Gtn-HPA and HA-Tyr.


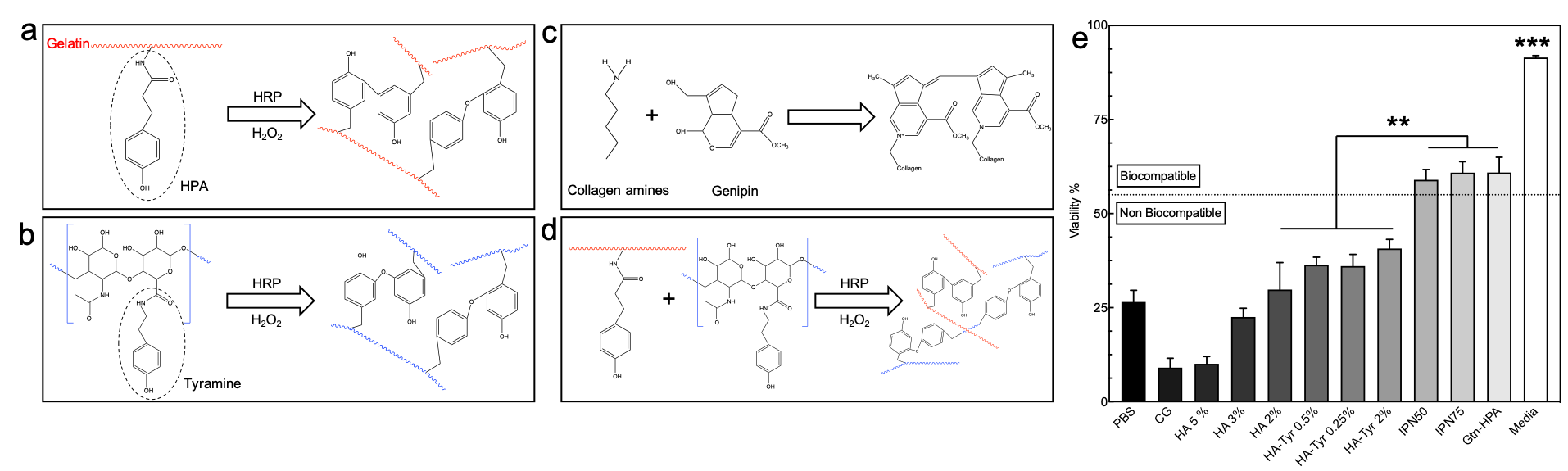


**Supplementary Figure 1. Chemical structures and biocompatibility assay of potential bioinspired hydrogels.**

Chemical structure and crosslinking reaction for Gtn-HPA (**a**), HA-Tyr (**b**), Collagen-Genipin (**c**) and IPN (**d**). Gelatin chemical structure is approximated with red-lines and hyaluronic acid backbone with blue lines. HPA and Tyramine groups are shown inside the dashed circles. **e.** Viability assay of human retinal ganglion cells in potential hydrogels to enable biocompatibility measurement. Biocompatibility threshold was placed at 55% (dotted line). Data shown as mean ± SEM of triplicate wells with 15 different fields for each well. One-way ANOVA, followed by Tukey’s test, was performed and shows a statistically high significant difference between media and all other groups (***p=0.0001), being our positive control. Significant increase of viability can be seen biocompatible hydrogels (IPN50, IPN75 and Gtn-HPA) compared to all others (**p=0.001).

## Analysis of IPN chemical structure (FTIR data)

Proving the formation of either a hybrid interpenetrating network (IPN) or random copolymer network was critical in terms of in vivo degradation kinetics and cell encapsulation. In the main text (Figure 1b) we show the in vitro enzymatic degradation kinetics for both homopolymeric networks Gtn-HPA and HA-Tyr and the IPN with different content of each polymer: ranging from 25% Gtn-HPA in IPN25 to 75% in IPN75. Enzymatic degradation by collagenase or hyaluronidase shows a percentage of mass loss being equal to the content of respectively Gtn-HPA or HA-Tyr in the IPN. This result suggests a strong crosslinks selectivity producing a hybrid IPN hydrogel. To confirm these findings, we performed Fourier transform infrared spectroscopy (FTIR) on all samples. The FTIR spectrum of the Gtn-HPA and HA-Tyr non-crosslinked solid polymers are shown in Supplementary Figure 2a.

A number of bands around 1390 cm^-1^ are attributed to the presence of type-I Gelatin ^1^, proving the provenance of our material. Hyaluronic acid presence is observed in the band at 1409 cm^−1^ which can be attributed to the stretching of COO^−^, referring to the acid group in the HA molecule ^2^. The absorption band at 1036 cm^−1^ is attributed to the linkage stretching of C-OH and finally, the stretching region of the protonated group COOH is observed at 1078 cm^−1^. The amide A band arising from N-H stretching was distributed at 3308 and 3277 cm^-1^, C-H stretching at ~2945 and 2912 cm^-1^ for the amide B, N-H deformation at 1539 and 1574 cm^-1^ for the amide II respectively for Gtn-HPA and HA-Tyr. C = O stretching at 1609 cm^-1^ for the amide I can be observed in HA-Tyr while the amide III can be seen at 1237 cm^-1^ for Gtn-HPA. The presence of HPA side group can be seen by the peaks at 1452, 1633 and 3085 cm^-1^ while Tyramine is visible at 1378 and 3085 cm^-1^.

To quantify crosslinking reactions between HPA and Tyramine groups in both homopolymeric networks and IPN we measured the FTIR spectra of dry gels (Supplementary Figure 2b). The appearance of a difference in transmission around 1000 cm^-1^ indicates the stretching of COO^-^ groups and therefore the presence of hyaluronic acid, which is shown to be increasing from IPN75 to IPN50 to HA-Tyr. Both side groups HPA and Tyramine have specific peaks respectively around 1600 and 1300 cm^-1^ in the non-crosslinked polymers. In IPN and hydrogels, an increase in transmission percentage can be seen for the HPA peak while a decrease is observed for the Tyramine peak from HA-Tyr to Gtn-HPA. Gtn-HPA doesn’t show any transmission around Tyramine peak but IPN transmission is relatively close to HA-Tyr. At HPA peak, HA-Tyr is significantly lower than all others while both IPN are close to Gtn-HPA. The similar transmission seen in homopolymeric networks side groups and IPN suggest a strong selectivity in crosslinks and the formation of a hybrid IPN.


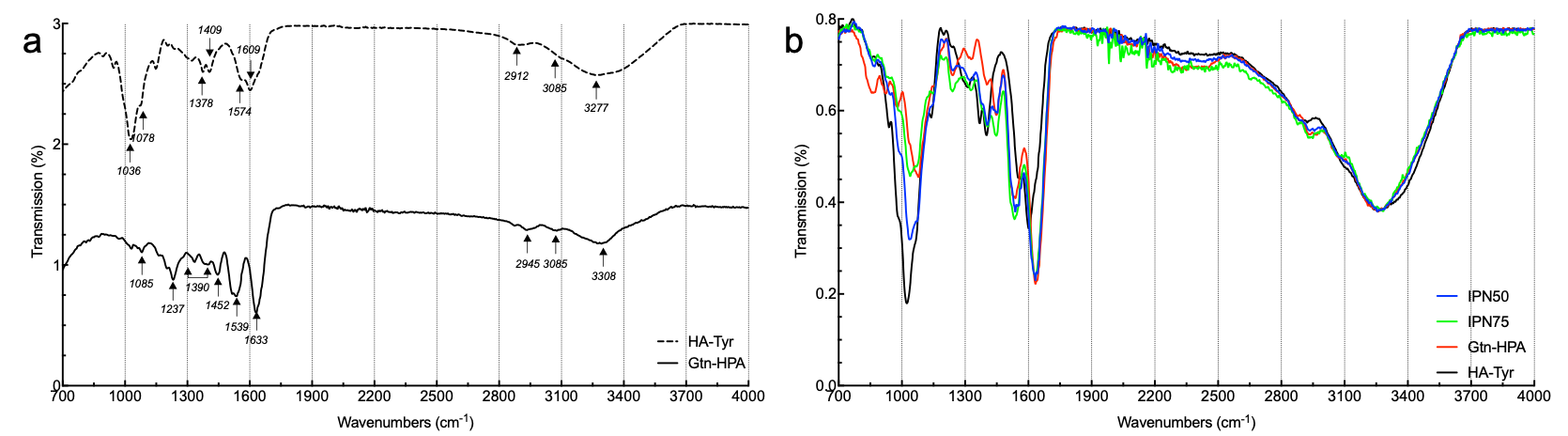


**Supplementary Figure 2. Fourier-transform infrared spectroscopy (FTIR) analysis of hydrogels and polymers.**

FTIR data of solid Gtn-HPA and HA-Tyr polymers (**a**), and dry hydrogels (Gtn-HPA, HA-Tyr, IPN75 and IPN50) (**b**) shown as baseline corrected and normalized transmission percentage in function of wavenumbers. For more visibility, polymer samples were spaced in the graph. Specific wavenumbers are marked with arrows and their value and normalized transmission percentage in function of wavenumbers.

## Mechanical properties measurement in hydrogels

Mechanical tunability of IPN was measured by oscillatory rheology and unconfined compression testing. Hydrogen peroxide, being cytotoxic to cells at high concentrations, has been shown to create biocompatible hydrogels around 1mM (see^3^ and Supplementary Figure 5a). This hinted at the mechanical measurement of HA-Tyr, Gtn-HPA and IPN at crosslinker concentrations equal or close to 1mM as seen in Supplementary Figure 3a. Shear moduli (G’) of both Gtn-HPA and HA-Tyr increase with crosslinker concentration ranging respectively from 160 and 1010 PA at 0.7 mM of H2O2 to 910 and 1650 Pa at 1.3 mM. A similar trend was observed for the time to reach steady state. This high difference could critically control a specific stiffness tunability by controlling the amount of Gtn-HPA in the IPN.

A similar trend was observed on the Young’s modulus, (Supplementary Figure 3b). HA-Tyr shows a plastic behavior at low strain (4.5%) with a modulus of 6818 Pa while IPN50, IPN75 and Gtn-HPA stay in the elastic domain for higher strain (8%).

In order to prove that gel point and stability of gels can be tuned by controlling the content of IPN we needed to be able to quantify the elastic nature of gels in the crosslinking process. G’ and G’’, as seen in Supplementary Figure 3c, measurements confirm the dependency of gel point on the Gtn-HPA content. Gel point ranges from 42s for Gtn-HPA, being far too quick for surgical needs, to 162s for IPN50. This is a crucial result as it confirms the hypothesis that these biocompatible IPN can be tuned to fulfill surgical needs.


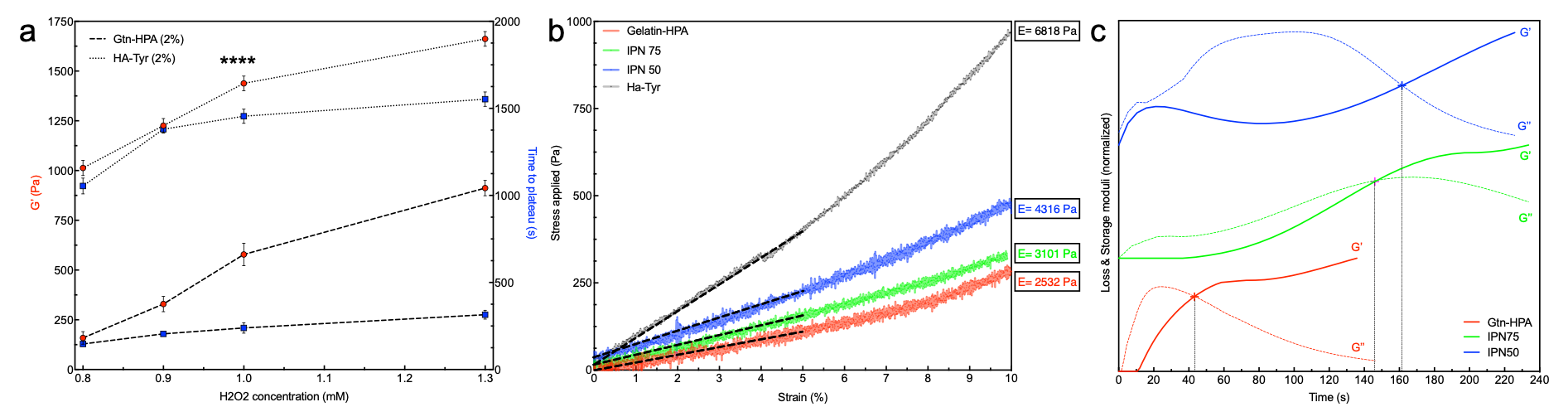


**Supplementary Figure 3. Hydrogels (IPN and homopolymers) rheological and mechanical characterization.**

**a.** Oscillatory rheological measurements of shear modulus (G’ in red) and time to reach plateau (in blue) for Gtn-HPA and HA-Tyr for different concentration of crosslinker H2O2 (ranging from 0.8mM to 1.3mM). Data shown as mean ± SEM of triplicate measurements. One-way ANOVA, followed by student-t test for each concentration, was performed and shows a significant difference in shear moduli between HA-Tyr and Gtn-HPA (****p<0.0001). **b.** Compression test measurements of Young modulus for Gtn-HPA, IPN75, IPN50 and HA-Tyr. Dashed line shows linear regression performed to measure moduli in the elastic region (strain less than 5%). **c.** Loss (G’’ dashed lines) and storage (G’ plain lines) moduli calculation from micro-rheological measurements for Gtn-HPA, IPN75 and IPN50 (see Supplementary Video 1 for more information). Gel point is represented with crossing of G’ and G’’ by vertical dotted lines.

## Molecular weights and transition temperature measurements

Complete characterization of solid polymers (Gtn-HPA and HA-Tyr), hydrogels and IPN was performed as seen in Table 1. Number (Mn) and weight (Mw) averaged molecular weight, glass transition temperature (Tg) and melting temperature (Tm) were measured with respectively gel permeation chromatography (GPC) and differential scanning calorimetry (DSC). As seen in Supplementary Figure 4a, a clear glass transition and melting transition were observed. However, differing from usual DSC experiments, both samples were destructured post melting and could not be brought back to initial shapes. Molecular weights (Supplementary Figure 4b and Supplementary Figure 4c) measures were fitted with a normalized gaussian curve showing higher molecular weight for HA-Tyr than Gtn-HPA. Some peaks were seen at really low weight (<5,000 g/mol) for both samples resulting from the presence of both HPA and Tyramine groups.


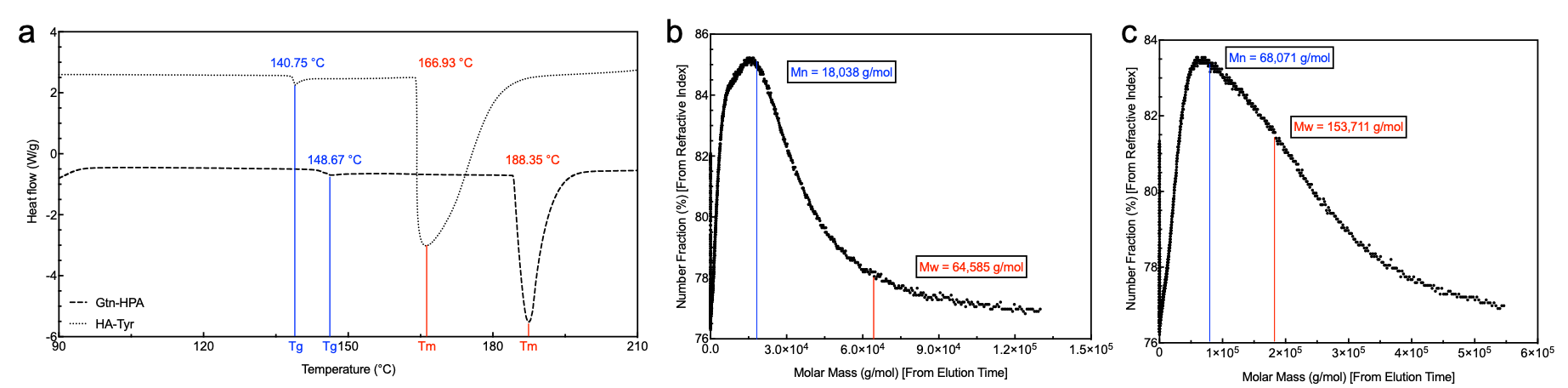


**Supplementary Figure 4. Transition temperatures and molecular weight characterizations of dry polymers.**

**a.** Differential scanning calorimetry measurements of solid Gtn-HPA (dashed line) and HA-Tyr (dotted line) polymers. Glass temperature (Tg in blue) and melting temperature (Tm in red) are represented with vertical lines with their values for both polymers. **b, c.** Gel permeation chromatography measurements of solid Gtn-HPA and HA-Tyr polymers. Number average (Mn in blue) and weight average (Mw) molecular weight were calculated (see methods) and represented on both graphs.

# Supplementary Note 2 - Distribution and characterization of cells in hydrogels

## Effect of H_2_O_2_ on cell viability and intensity measurement

One of the most impactful stress that appears on cells encapsulated in Gtn-HPA and HA-Tyr is oxidative stress due to the presence of hydrogen peroxide as a crosslinker (usually cytotoxic to cells in high doses). We performed a viability assay on cells encapsulated in hydrogels with increasing concentration of H_2_O_2_ to find the optimal formulation for both homopolymeric networks: this could then be transferred to IPN (Supplementary Figure 5a). For all samples (Gtn-HPA and HA-Tyr at different wt%) crosslinked with H_2_O_2_ around 1mM seemed to provide the highest biocompatible hydrogels with viability ranging from 35% in HA-Tyr at 0.5 wt% to 60% for Gtn-HPA at 2 wt%. Of note is that we observed, for low concentration of H2O2 (<0.8mM), almost no gel formation which is shown by a lower viability (similar to culture in PBS) at 0.5mM. Finally, we increased hydrogen peroxide concentration to 2.5 and 5 mM in order to look for its cytotoxic effect on cells. Oxidative stress was already really high at 2.5 mM with a viability ranging from 20% to 35% while being maximal at 5mM where most cells died (only 5-8% viable). This broad testing of hydrogen peroxide effect on cell viability in both homopolymeric networks suggested that a concentration around 1mM should be used in order to make the most biocompatible hydrogel. As seen in Figure 2a, we performed a sharper testing for different IPN content with crosslinker concentration ranging from 0.8 to 1.3 mM. The optimal IPN was found to contain at least 30% of Gtn-HPA with a crosslinker concentration of 1mM. As seen in past studies ^4^, the catalyst (HRP) concentration was already optimized at 0.1 U/mL to enable encapsulated cells to thrive.

Cells in 2D culture, in Supplementary Figure 5b, appeared more fibroblastic with a higher number of dead cells. In contrast, cells encapsulated in Gtn-HPA and IPN seemed to retain their phenotypic morphology. Our result clearly demonstrates the effect of oxidative stress and protective nature of Gtn-HPA and IPN for hRGC culture and possible transplantation.


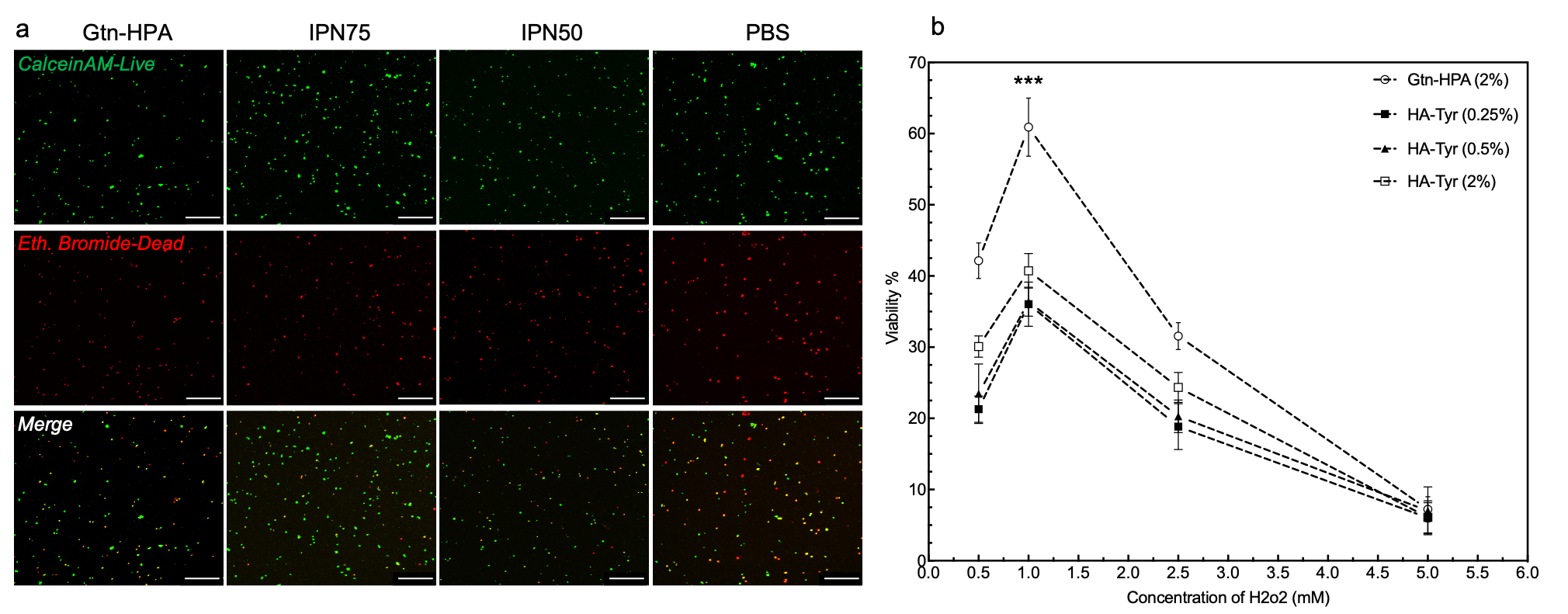


**Supplementary Figure 5. Encapsulated human retinal ganglion cells viability assay.**

**a.** Fluorescence images of live (CalceinAM-green) and dead (Ethidium Bromide-red) hRGC encapsulated in hydrogels, maximum projection of 300 um samples imaged with confocal microscopy. All images were taken at 10X magnification under fluorescence microscopy. Scale bar - 200um. **b.** Viability quantification of human retinal ganglion cells in Gtn-HPA and HA-Tyr (0.25, 0.5 and 2%wt) with different crosslinker concentration (hydrogen peroxide ranging from 0.5mM to 5mM). Data shown as mean ± SEM and one-way ANOVA followed by student-t test was performed showing a statistically high significant difference between Gtn-HPA and all others for H_2_O_2_-1mM (p=0.0001).

## Analysis of cells size and shape with image processing algorithm

Counting and analyzing cell size and shape manually is a tedious error prone process. To be able to analyze a large data set from fluorescent confocal microscopy images, we decided to use a basic computer vision technique. This image processing algorithm, previously explained in^5^, enables for cell segmentation from a black background image based on cell intensity and staining. The method uses basic morphological operations and the watershed algorithm to segment the cells and was implemented in MATLAB. We chose this method for its simplicity and ease of implementation (see Methods for more information).

In the sample image 33 cells were extracted (Supplementary Figure 6f). We also extracted cell size (Supplementary Figure 6g) and the shape factor (Supplementary Figure 6h). Segmenting grouped cells worked well and enabled a coherent cell size and number measurement. Detected cells appear to have a similar size to real cells (broadly measured with scale on confocal microscope). By counting some fields of views manually and comparing to the algorithm result we can suppose that this algorithm has a rate of success of around 95%.


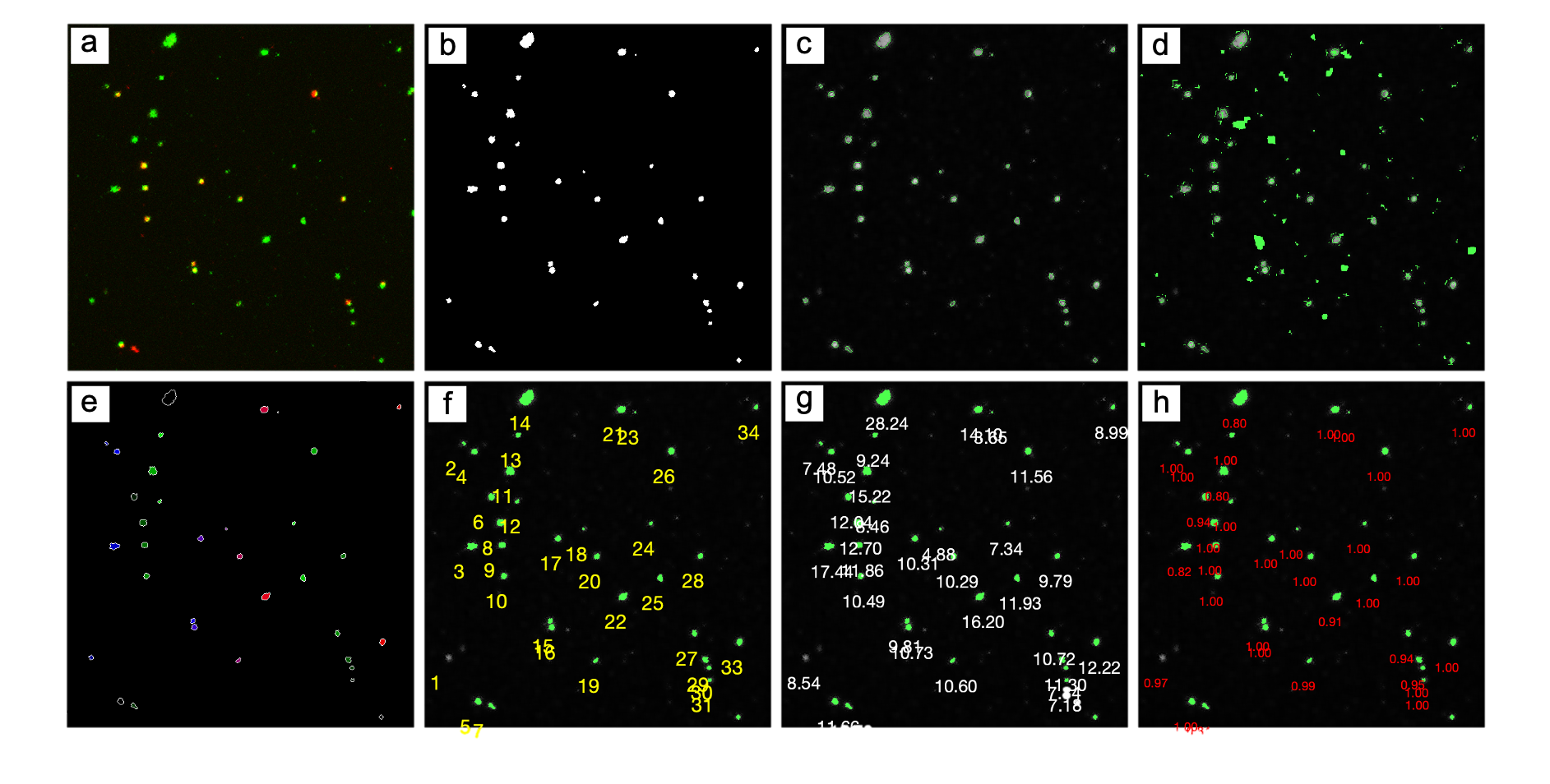


**Supplementary Figure 6. Image processing algorithm for measuring cell size and shape.**

Step by step image processing analysis of live and dead stained hRGC encapsulated in hydrogel or in 2D conditions. **a.** Initial maximum projection fluorescence image taken with confocal microscopy at 10x magnification. **b.** First processed image after contrasts adjustments, elimination of objects on the borders, noise removal and threshold with Otsu’s method. **c.** Perimeter cell extraction. **d.** Image processed with watershed algorithm to separate possible grouped cells. **e.** Connected components from the watershed results. **f.** Number of cells extracted per image. **g.** Cell size for each object. **h.** Cell shape factor corresponding to its shape compared to a perfect circle.

We applied this image processing algorithm to the long viability assay performed on hRGC for 1, 3 and 7 days in different conditions (see Figure 2b in the main text). All extracted values form the image processing algorithm can be seen in Supplementary Figure 7 for all samples. Live (green) and dead (red) cell size distribution was analyzed for all groups and shows no significant difference in time. The results suggest that dead cells are, on average, 10 um smaller than live cells. A significantly higher live cell size was found in hydrogel samples (around 15um for Gtn-HPA, 20 um for IPN75 and IPN50) compared to cells cultured in 2D environment (8-10um for media and PBS). The shape factor was also analyzed for both live and dead cells for all samples and shows no difference between time points. A significantly lower shape factor was observed for cells in hydrogels samples compared to media and PBS. Dead cells, mostly due to the loss of cytoplasm, nucleus and shape, show a low shape factor for all samples. These findings suggest that Gtn-HPA and IPN can enable cell growth and differentiation due to their higher size while enabling morphological extension of primary processes, as shown with a lower shape factor.


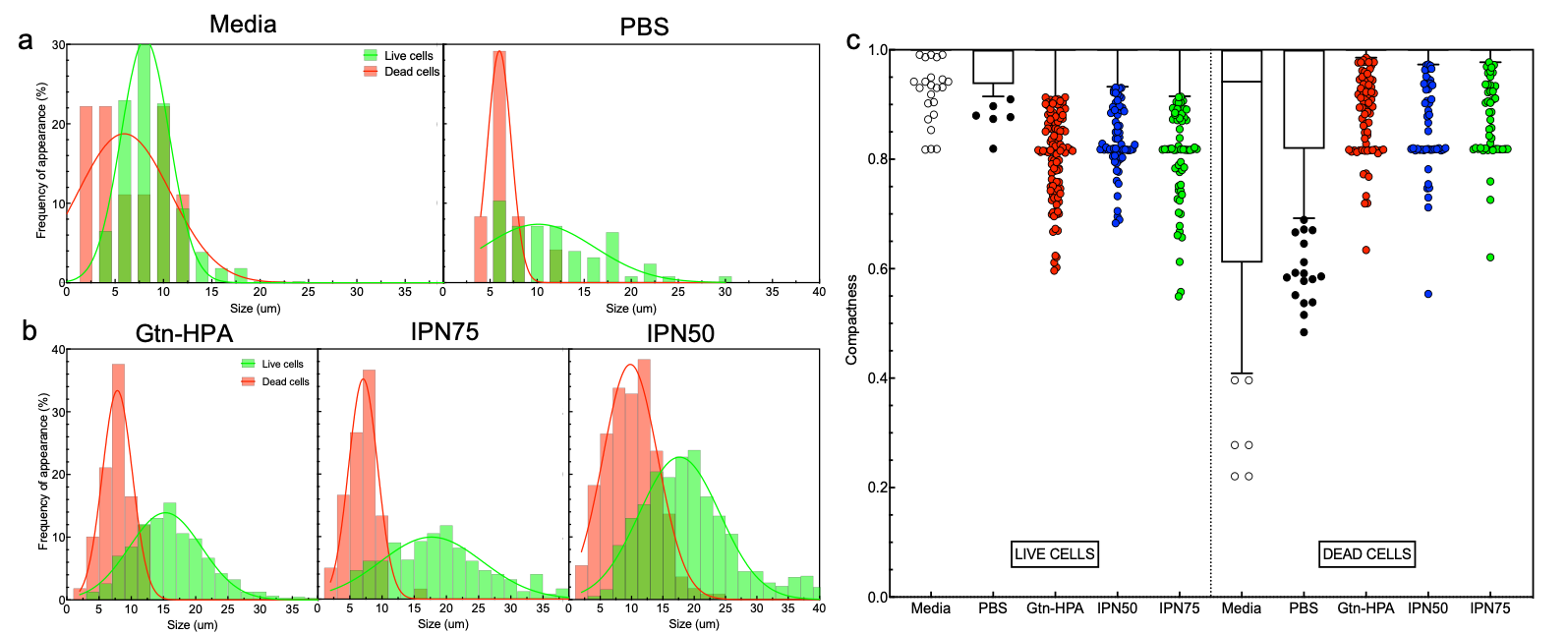


**Supplementary Figure 7. Human retinal ganglion cells size and shape quantitative analysis.**

Live and dead cell size distribution for hRGC cultured in 2D conditions (media and PBS, **a**) or in hydrogels (Gtn-HPA, IPN75, IPN50, **b**). **c.** Live and dead cell shape factor quantification for all samples. No statistically significant difference was found in this analysis. Data shown as boxplot with central line for average, bound of boxes for 75% interval and whiskers for SEM.

## Immunohistochemistry and flow cytometry assay

Phenotype was checked by flow cytometry and immunohistochemistry on cells cultured for 5 days in 2D (media) or 3D (Gtn-HPA, IPN75 and IPN50 with media) conditions. Our hRGC phenotypic expression was measured in previous studies^6^ and was shown to have a high positive cell population expressing early RGC markers (such as Brn3a) with a moderate population of late retinal ganglion cells. The gating strategy for flow cytometry is presented in Supplementary Figure 8. And consisted in: gating the cell population with FSC-SSC, then gating the single cell population with the linear representation FSC-H/FSC-A and finally gating the DAPI positive population in VioBlue-FSC. This final population was then analyzed for each isotype control where the gate was put at 2.5% and pasted on all other markers.

**
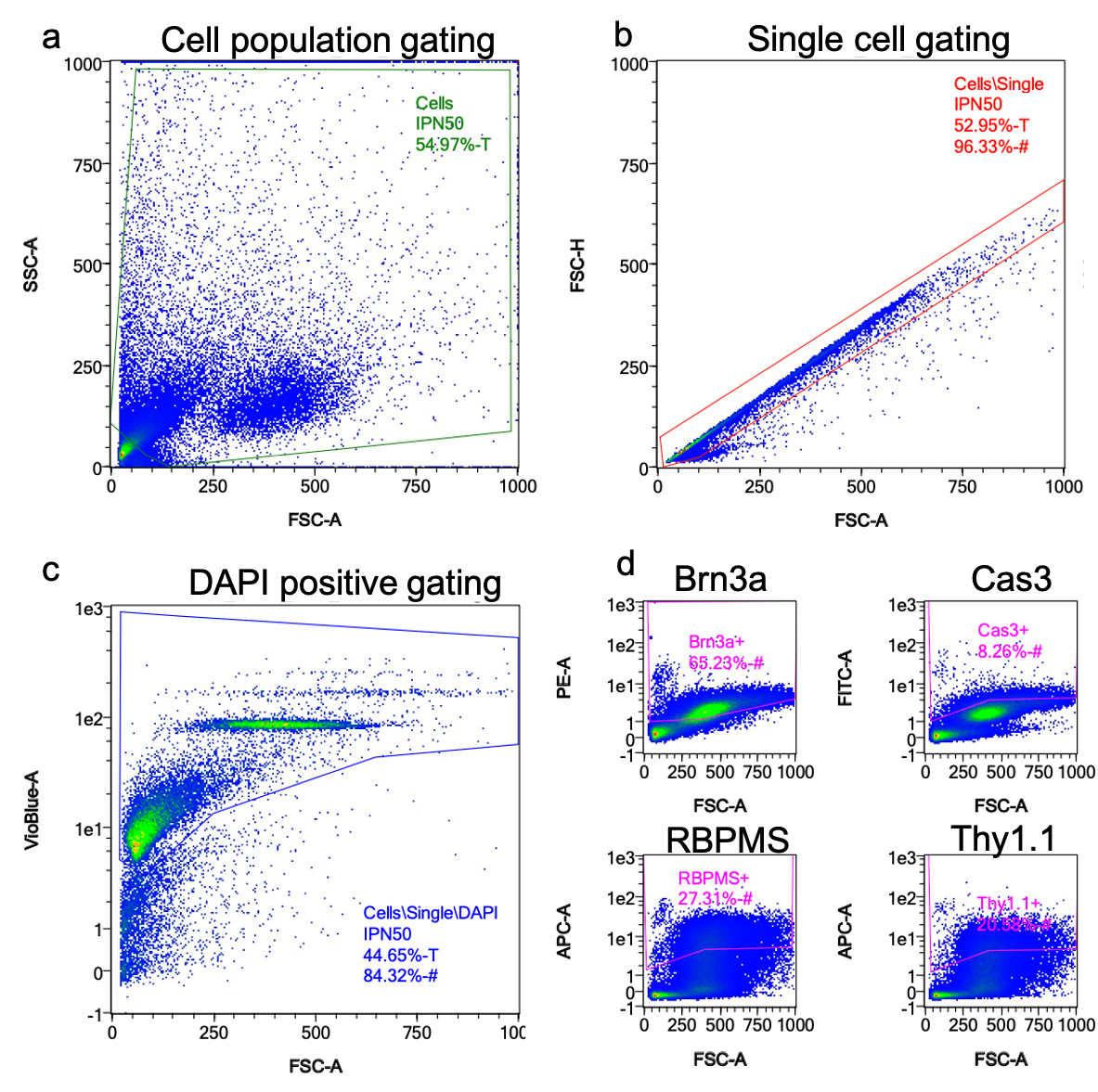
**

**Supplementary Figure 8. Flow cytometry gating strategy for hRGC.**

Gating strategy for flow cytometry performed on hRGC to check their phenotype. **a.** Cell population gating on SSC-A/FSC-A channels. The cell population was marked for all events in the middle region of the channels (removing small debris and large artefacts. **b.** Single cell population gating on FSC-A/FSC-H channels. The single cell population is marked for events having a linear correlation between height and area. **c.** DAPI positive population on VioBlue-A/FSC-A. Only positive DAPI events are marked. **d.** Examples of markers gated (RBPMS, Brn3a, Thy1.1 and Cas3). Gates are placed at 2.5% on isotype controls and then applied on all other markers.

As explained in previous study^7^, our hRGC are labelled and isolated with intrinsic Brn3B-TdTomato expression, this result in a high expression of Brn3B in phenotypic analysis. Percentage of expression of retinal ganglion cells (Brn3b, Brn3a, RBPMS and Thy1.1), neuronal (NeuN), apoptosis (Cas3), proliferation (Ki67) and stemness (Cmyc and Oct4) was measured and is reported in Supplementary Figure 9a while actual cell expression is shown with confocal microscopy fluorescent images (Supplementary Figure 9b). Images were taken at field of views presenting a high expression of each marker and are not representative of overall marker percentage of expression. Of note is that a critical factor of using a scaffold to encapsulate cells is to ensure the maintenance of phenotype throughout the entire experiment.

Stemness, proliferation and apoptosis expression were remarkably low in all samples. This suggests an already differentiated cell population (with low number of pluripotent stem cells) which is past mitosis. A low apoptosis expression in all samples enables us to consider the hydrogels non-toxic and confirm the viability assay performed on all IPN with healthy cells in 2D and 3D environments.


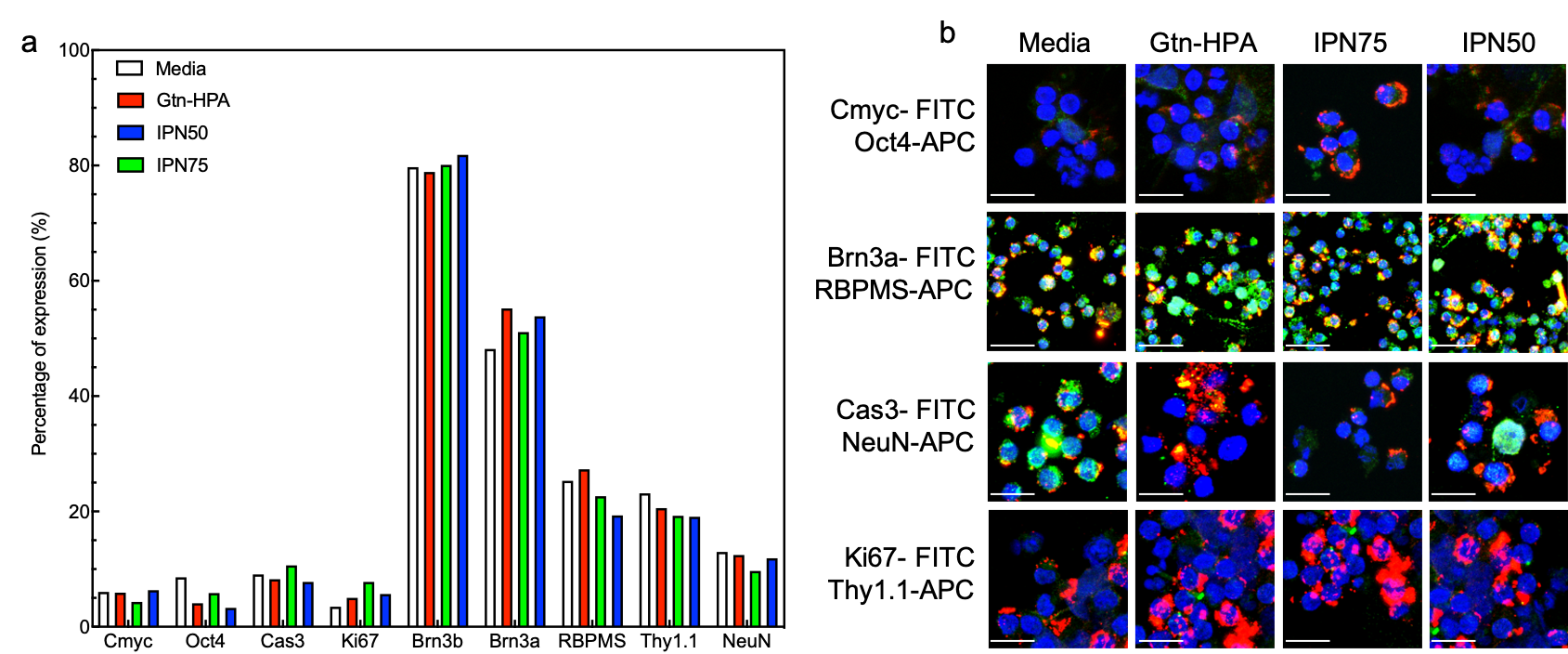


**Supplementary Figure 9. Human retinal ganglion cell immunohistochemistry and flow cytometry assays.**

**a.** hRGC cultured in media, within 2%wt Gtn-HPA, IPN75 or IPN50 for 5 days were analyzed using fixed flow cytometry assay. Isotype control mouse and rabbit were gated at 2% and are not represented in this graph. Percentage of positive hRGC which express stemness markers (Cmyc and Oct4), apoptosis and proliferation markers (respectively Cas3 and Ki67), ganglion cells markers (Brn3b, Brn3a, RBMPS and Thy1.2) and neuronal markers (NeuN) is presented for each sample. **b.** Fluorescence images of markers analyzed with flow cytometry, with DAPI as nuclear staining (VioBlue channel) for all samples (Media, Gtn-HPA, IPN75 and IPN50). Merged images only. Scale bar is 40 um in all images. Images taken under fluorescence microscopy 63X magnification.

As explained in Method, most cells being Brn3b-Tdtomato positive (due to the isolation process), Brn3b expression was primordial to confirm cells phenotype maintenance by hydrogels and protocols. This expression is critically high for all samples (around 80%) which also indicate the possibility of locating injected cells in tissue without the need of many staining. Brn3a is a marker which overlaps with Brn3b and is shown here to be relatively high for all samples (around 50%) indicating a high percent of early retinal ganglion cells in the population. RBPMS and Thy1.1 are markers for late retinal ganglion cells, already fully differentiated. Part of the hRGC population (around 20%) is expressing those two markers, suggesting cells differentiated already present.

Finally, retinal ganglion cells’ main objective is to extend processes (axons) that merge into fibers to form the optic nerve. These fibers express some neuronal markers such as NeuN. A small part of the hRGC population already expresses NeuN after 5 days in gels (10%) which suggest that injected cells could potentially be able to extend processes that could attach the retina and regenerate a dying RGC layer. This assay enables us to confirm the maintenance of hRGC phenotype when encapsulated in IPN with nutrients for 5 days in vitro.

# Supplementary Note 3 - Gel attachment to the retina

## Gel-ILM interface analysis with H&E staining

To prove if our IPN could be mechanically tuned in order to make contact or attach with the back of the eye (inner limiting membrane of the retina: ILM) we injected Gtn-HPA, IPN75 and IPN50 in the vitreous of Long Evans rats. H&E staining showed gel presence on top of the retina for all samples.

The percentage of attachment was calculated for each portion and summed over all replicates which gave for Gtn-HPA, IPN75 and IPN50 respectively 50%, 59% and 79%. By summing over all replicates, we were also able to obtain the actual distribution of attachment which suggests the presence of holes at the interface (see Figure 3b). These findings suggest that a higher stiffness and mechanical strength (as in IPN50) could enable a better attachment of injected hydrogels to the retina: higher average attachment and less holes at the interface. When injected cells in saline in the vitreous, cells don’t have matrix to stay at the site of injection (usually ILM or RGC layer) and can potentially leave the site and start the process of apoptosis, not being able to attach to other cells. Using these tunable IPN we can encapsulate cells and inject them at the interface where stiffer hydrogels could then attach and release cells directly onto the targeted layer of the retina.

Of note is that we performed H&E staining 1- and 10-days post injections. In the first case gel was not fully attached to the eye but was present in the vitreous cavity, while in the latter most gel was already degraded without any detachment of the retina or its layers.


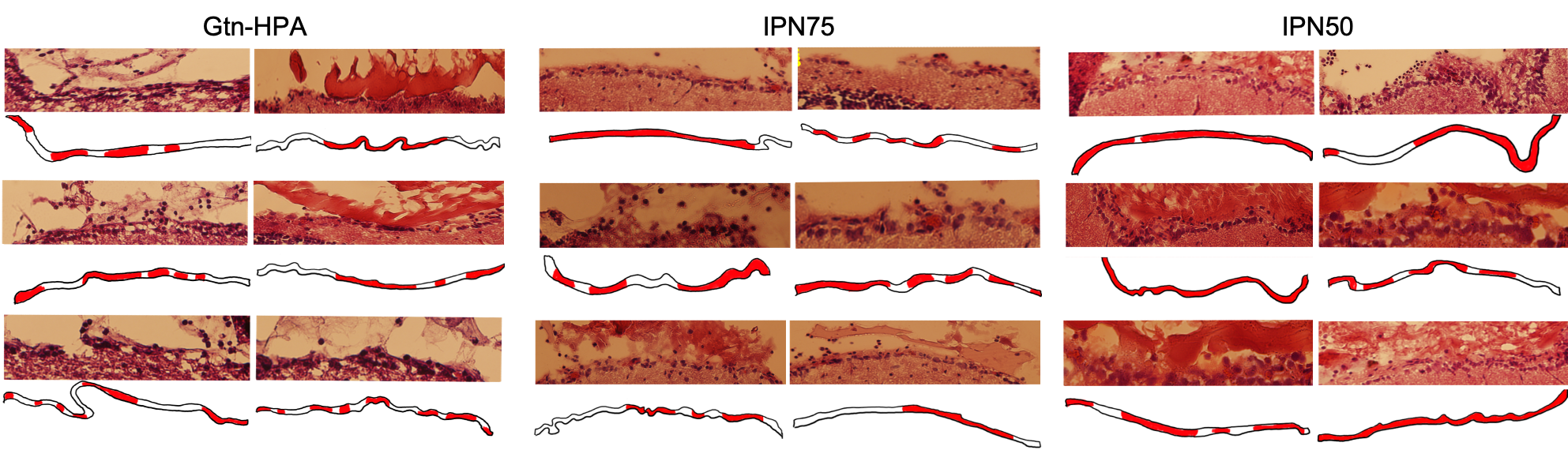


**Supplementary Figure 10. H&E staining and hydrogel-ILM interface analysis after injection of gel in Long Evans rats.**

H&E stained 200um sections of the retina, centered on the interface hydrogel-ILM for all groups. Analysis of attachment shown below each image with black lines delimiting the interface and red regions showing attachment of gels to the retina.

## Optical coherence tomography data

When injecting in vitreous Gtn-HPA, IPN75 and IPN50 we performed live Spectral Domain Optical Coherence Tomography (SD-OCT) to image the back of the eye with infrared (IR) and sections of the retina without sacrificing animals. We were able, for the first time, to image and observe vitreal injected gel live in vivo. As seen in Supplementary Figure 11, we acquired images of the back of the eye showing healthy blood vessels, the core of the vitreous and multiple sections of the retina before, after and each day post-injection.

Due to the size of the animals, and the SD-OCT apparatus not made for rodents the quality of SD-OCT images were lower than expected. However, we still observed the presence of islands of gels present in the bulk of the vitreous right after injection for all samples which implied a difficult imaging of the section of the retina at that time point. Indeed, islands of gels were refracting the OCT light far away from the retina therefore sectioned looked cut in half. This will be mainly solved by waiting for the setting of the gel on top of the retina. These islands are characteristics of Gtn-HPA and HA-Tyr in-situ crosslinking hydrogel formation^8^ and suggest a successful injection. While the back of the eye was still blackened by the gel, its islands were observed to be sitting on top of the retina around 2-3 days for all samples. White regions of gels attached to the retina suggest 3 days to be the optimal time for setting and start of degradation. We observed a drop-in gel presence starting at 4, 5 and 6 days respectively for IPN75, Gtn-HPA and IPN50. This finding was reinforced by the total absence of gel for all samples after specific time marked on Supplementary Figure 11 (8-9 days for IPN50, 6-7 days for Gtn-HPA and 4-5 days for IPN75). One crucial finding is that morphology of the retina and the back of the before injection and after total degradation of gel are similar, showing no sign of retinal injury nor detachment. These findings suggest successful injection of gel that is enabled to sit on top of the retina and degrades at a moderately fast rate without provoking retinal detachment.

To quantify these findings, we analyzed SD-OCT data with an image processing (See Method). This enabled us to partially quantify the presence of hydrogel in the eye in vivo throughout time (see Figure 3c). We then compared this quantification with a long-term in vitro degradation assay by using degrading enzymes (hyaluronidase and collagenase) with their actual concentrations found in vivo in the vitreous ^9,10^. Both in vitro and in vivo data show similar trends with a high correlation coefficient (R^2^=0.97) which suggest a correct analysis and measurement of gel presence in vivo with SD-OCT.

**
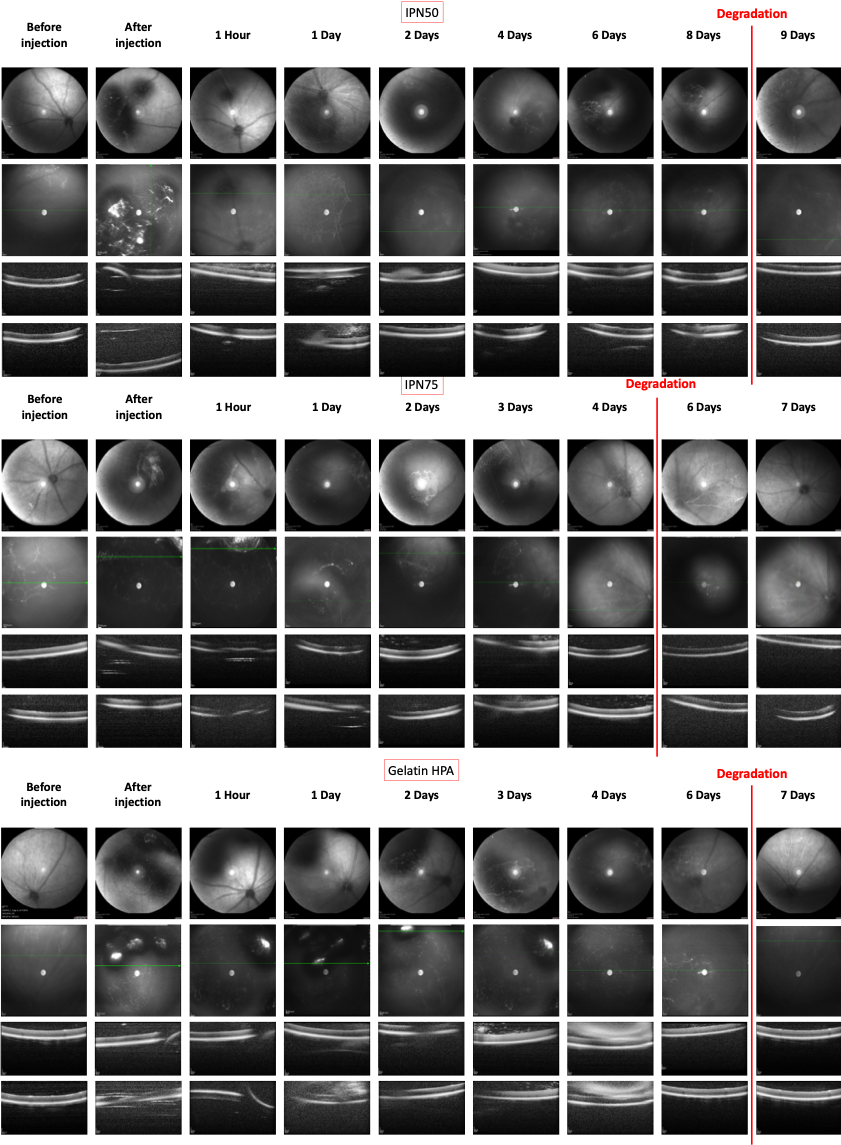
**

**Supplementary Figure 11. Optical coherence tomography and back of the eye imaging of Long Evans rat’s post-injection.**

**First row:** back of the eye imaging of rats injected with hydrogels (Gtn-HPA, IPN75 and IPN50) 3-days post injection. **Second row:** SD-OCT view of the section imaged. **Third and fourth rows:** SD-OCT sections imaging of live retina in rats injected with hydrogels showing retinal layers and presence of gels. Approximate degradation time is marked as a red line between two data points. Red arrows point towards islands and presence of gel on the retina and in the vitreous.

# Supplementary Note 4- In vivo imaging and analysis

## Confocal imaging of retina sections containing injected cells

As seen in Supplementary Figure 12a, STEM121 positive cells were observed next to the optic nerve for IPN50 and IPN75 while being in the center of the retina for Gtn-HPA and sparsely distributed everywhere for the PBS sample. Due to its low intensity and the size of tiling, intrinsic TdTomato-Brn3b was observed to be really dimmed in all tiling. These tiling suggest the success of our xenotransplantation but also of engraftment due to the long-term experiment. Indeed, due to the fast degradation of hydrogels injected cells had to attach and integrate to living tissue in order to thrive for 1 month in vivo. These tiling also enabled us to measure the size of the retina and by using an image processing algorithm (explained in the next section) to calculate the exact fraction of injected cells (5x10^4^ per eye) engrafted after 1 month for each whole eye.

To further analyze location and engraftment of injected human retinal ganglion cells, we imaged sectioned at 20x magnification for all samples (Supplementary Figure 12b). Cells were found to be mainly in the retinal ganglion cell (RGC), which is the targeted layer, and the inner nuclear layer (INL) some migrated towards the choroid, stopping in the outer nuclear layer (ONL). On first sight, we observed that cells in stiffer hydrogels (IPN5) and IPN75) were larger and more abundant than the one in Gtn-HPA. Overall cells injected in PBS were found to have the smallest size and were really low in number throughout all sections analyzed. Compared to tiling, intrinsic TdTomato-Brn3b was observable in these images and shows coherent location with injected cells (co-localization of both markers being analyzed in the next section).

To understand and explain why injected hRGC, in the vitreous, migrate towards another layer of the retina, one needs to look at the difference in size and morphology due to the experiment being a xenotransplantation. As a fact, human RGC are 2-3 times bigger than rats RGC ^11^, therefore, even being attracted to stay and engraft on the RGC layer, those cells try to find the right place to thrive by migrating into other layer with bigger cells (as in INL or ONL). Of note is the location of most cells being in the targeted layer (RGC) for hydrogels groups suggesting a better release of cells throughout time onto the retina compared to cells injected in PBS only.


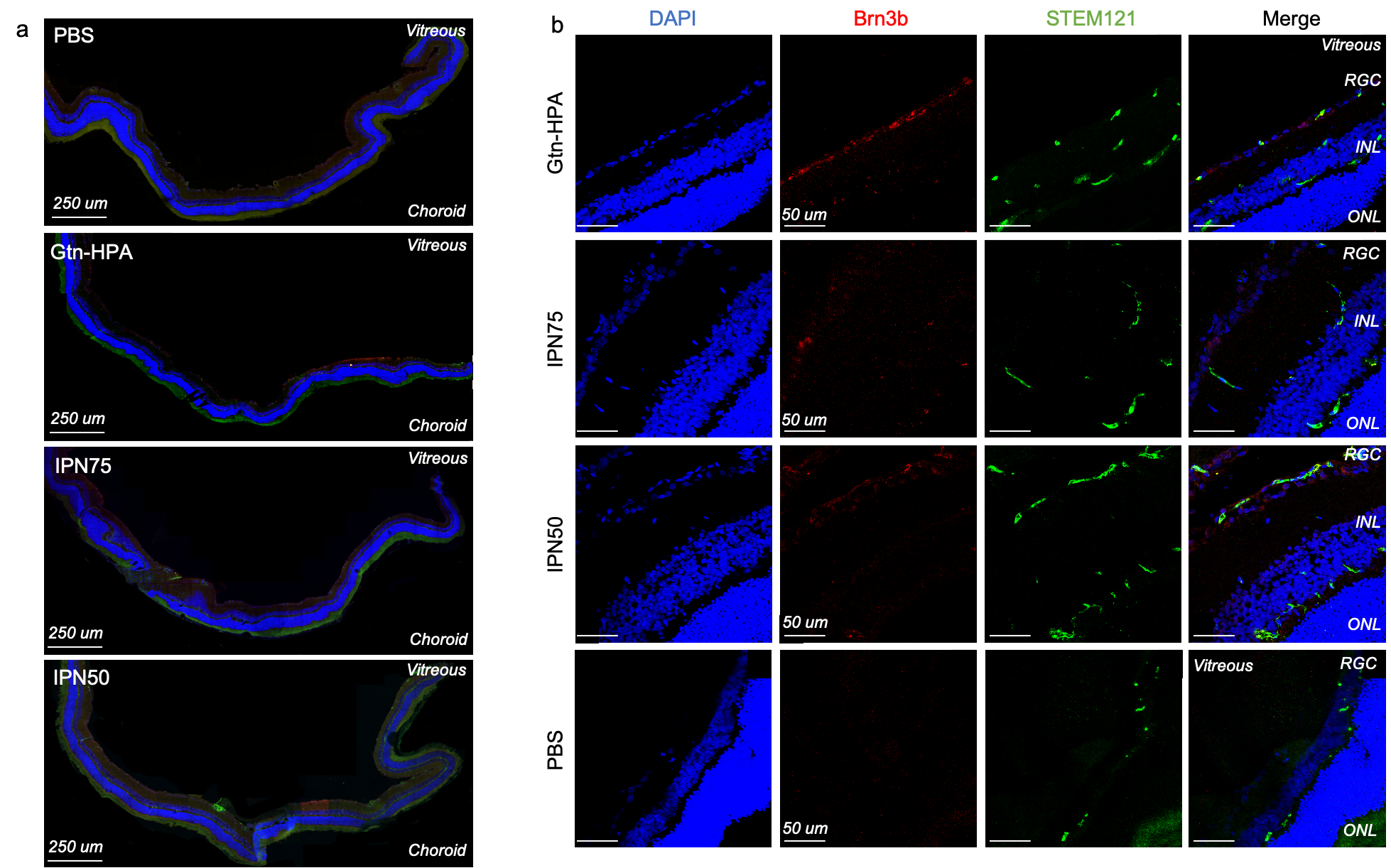


**Supplementary Figure 12. Imaging of hRGC 1-month post transplantation in Long Evans rats.**

Fluorescence microscopy images of retina 1-month post vitreal xenotransplantation of hRGC encapsulated in IPN to identify presence, location and engraftment of human ganglion cells in the host retina. Slides were stained with DAPI-VioBlue (for retinal structure and nuclei staining), STEM121-FITC (human marker) and Brn3b-TdTomato (intrinsic marker expressed in all injected hRGC). **a.** Tiling of the retina for the 4 injected groups. Images were taken at 63X magnification with confocal microscopy (scale bar – 250 um). **b.** Single channel fluorescence images, taken at 20X magnification, of hRGC injected in all groups (Gtn-HPA, IPN75, IPN50 and PBS). Last column shows merge image with retinal layers labelled. Scale bar is 50 um for all images.

## Cell migration, orientation and co-localization algorithms

Most stem cells in vivo transplantations are usually analyzed qualitatively by observing stained sections and cell morphology without applying quantification processes. To enhance this analysis, we created an image processing algorithm capable of localizing cells in their respective layer of the retina, calculating their relative orientation compared to the tissue and measuring the amount of both markers (STEM121 and Brn3b) co-localization in the cell body.


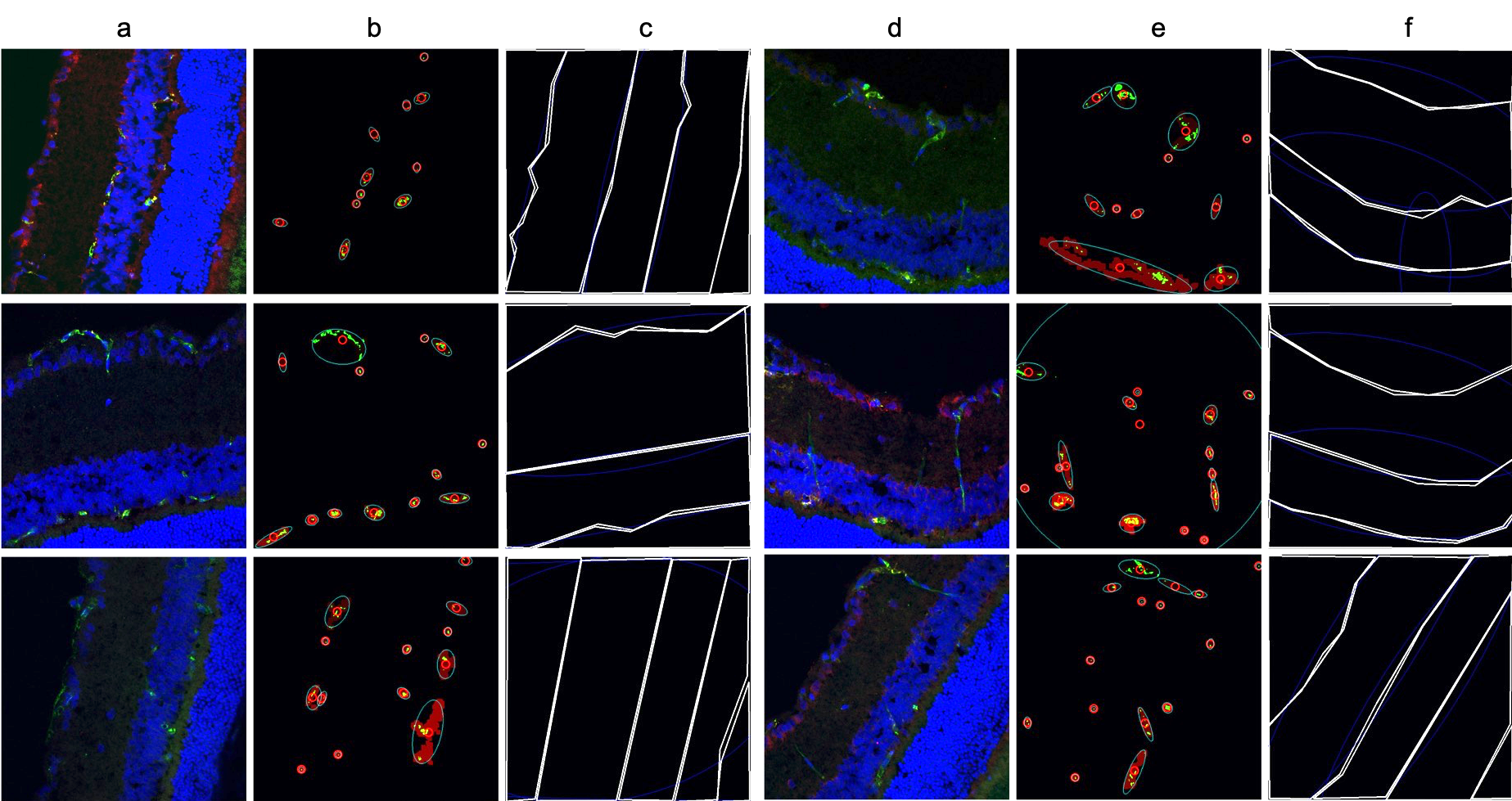


**Supplementary Figure 13. Image processing algorithm for measuring in vivo cell migration, orientation and co-localization.**

**a, d.** Learning data set of fluorescence microscopy images of retina, stained with DAPI (blue) and STEM121 (green) taken at 63X magnification. High contrast and thresholding methods were applied to analyze and quantify these images, implying over saturation of markers. **b, e.** 2 steps thresholding process images to capture cell center (represented by red circles), cell bodies (red and green regions) and cell orientation (blue ellipse stacked on cells). **c, f.** Images annotated and separated into the different retinal layers (regions seen as white geometrical shapes).

As explained in Method the algorithm consisted in a 2-step thresholding process which separated the cell center (possessing a high intensity) from their surrounding (dimmer intensity). Nonetheless, being able to extract cell location on each image was not sufficient to significantly quantify our xenotransplantation, therefore we decided to annotate each image with the retinal layers as seen with DAPI staining (third column): dividing each image into retinal regions (Choroid, ONL, INL, RGC and Vitreous).

## Muller and immune cells expression

While in vivo xenotransplantation was performed on immunosuppressed Long Evans rats, analyzing and measuring the core immune response to injected cells and biomaterials is critical to any transplantation. To do so, we stained eyes sections with IBA1, CD45, CD11b and CD68 markers (mainly expressed in microglia and immune cells response to injection ^14^ in the retina). Most IAB1 expression was found in the inner nuclear layer (Supplementary Figure 14a) while CD45 was localized next to the injection site in the retinal ganglion cells layer. As seen in Supplementary Figure 14b, both immune markers expression was significantly higher in PBS than all other samples. In order to extract the effect of the gels from the injection itself we compared this data to the SHAM experiment (consisting in only stabbing the eye with the needle without injecting cells or buffers). All hydrogel samples possess a similar expression of immune cell markers compared to SHAM but higher than the control eye.


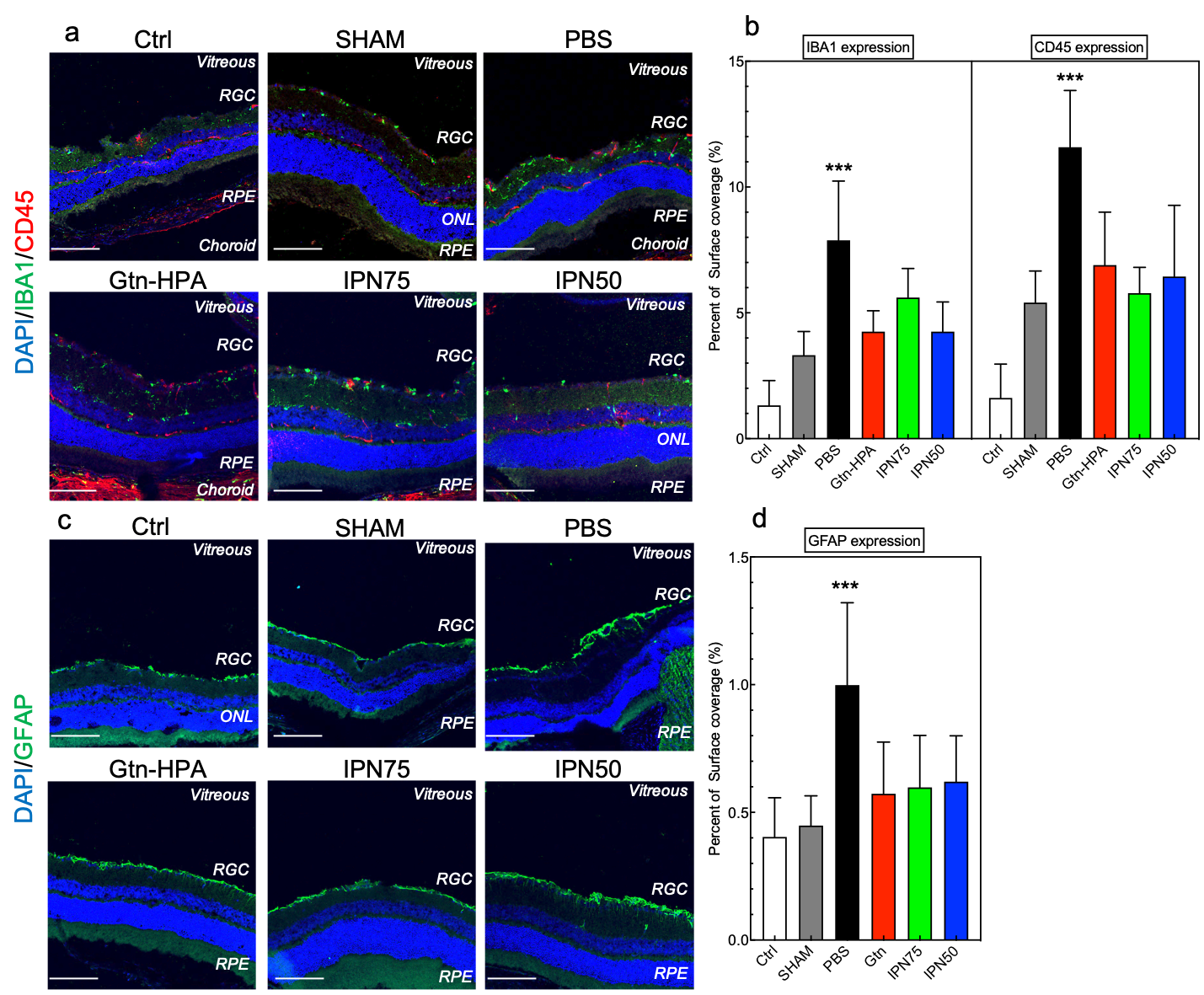


**Supplementary Figure 14. Immune response staining of xenografts, 1-month post transplantation.**

Images taken under fluorescence microscopy to identify leukocytes, immune cells and Muller cells expression. All images were taken at 63X magnification. Immunohistochemistry staining of all groups (Control, SHAM, PBS, Gtn-HPA, IPN75 and IPN50) for **a.** CD45 (APC) for leukocytes, IBA1 (FITC) immune cells or **c.** GFAP (FITC) for Muller cells activation. DAPI (VioBlue) staining was performed on all slides to provide retinal structure. Retinal layers were labeled on all images and scale bar is 100 um. **b, d.** IBA1, CD45 and GFAP expression, obtained from number of colored pixels, analyzed using one-way ANOVA (***p=0.001), shows a significant difference in surface coverage percentage between PBS and all other groups for all three markers. All data is shown as mean ± SEM.

Specific immunolabeling of activated microglial cells was analyzed with anti-CD11b, anti-CD68 (Supplementary Figure 15) antibodies revealed high expression in group with PBS injected cells and significantly lower in the group that received cells with gels for CD68 while no significant difference were found for CD11b. Innate immune response usually mounts early in response to stress, infection, and injury and since cells with gel have shown to cause less stress during transplantation the significant lower activated microglial response was noted in group that received cells with gels.

These findings suggest that the main cause of immune response when injecting cells encapsulated in our IPN is the needle injection itself, while injecting cells in PBS provokes a higher immune response. The immune reaction analysis showed the injury due to the injection could trigger a reaction from the host as seen with CD45 staining. In the PBS group, hRGC suspension are exposed to the invading leukocytes especially as xenograft in non-immunosuppressed animals. Gtn-HPA and HA-Tyr, being GMP-like approved biocompatible and biodegradable polymers, can form a protective barrier for hRGC, protecting them from the migrating immune cells.


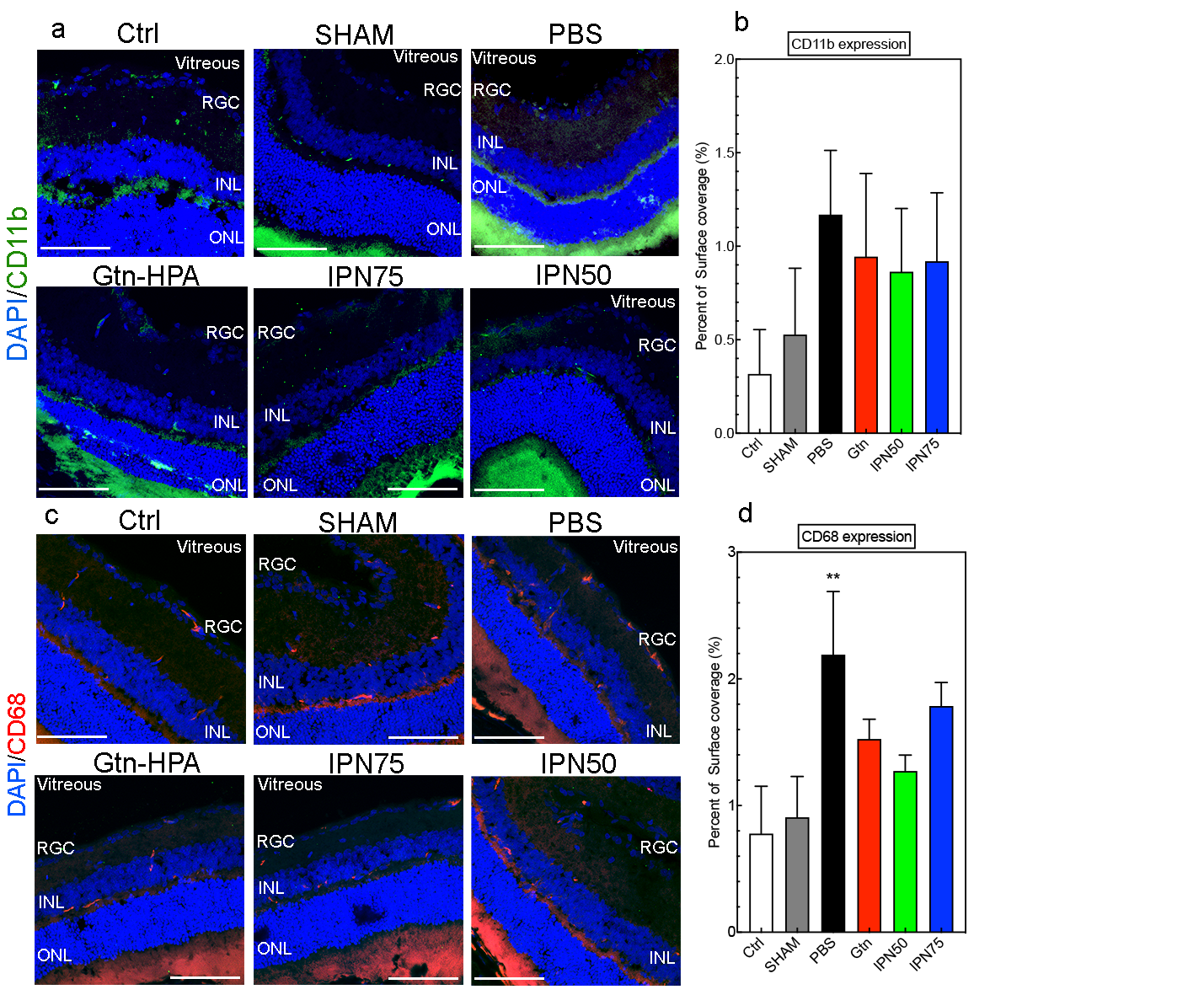


**Supplementary Figure 15. Activated microglia response staining of xenografts, 1-month post transplantation.**

Images taken under fluorescence microscopy to identify activated microglia, expression. All images were taken at 63X magnification. Immunohistochemistry staining of all groups (Control, SHAM, PBS, Gtn-HPA, IPN75 and IPN50) for **a.** CD11b (FITC) or **c.** CD68 (APC). DAPI (VioBlue) staining was performed on all slides to provide retinal structure. Retinal layers were labeled on all images and scale bar is 100 um. **b, d.** CD11b and CD68 expression, obtained from number of colored pixels, analyzed using one-way ANOVA (**p=0.005), shows a significant difference in surface coverage percentage between PBS and all other groups for CD68 marker. All data is shown as mean ± SEM.

Muller cells form the entrance path inside the retina, extending their processes through all the layers partly forming the barrier between the retinal ganglion cell layer and the vitreous: the inner limiting membrane. When activated, due to entrance of pathogens, migrating immune cells or exogenous ed human cells, Muller cells express largely Glial fibrillary acidic protein (GFAP). To measure Muller cells activation, we stained eyes sections with GFAP, as seen in Supplementary Figure 14c. An identical trend, as for immune reaction , was found in Muller cell activation with a significantly higher expression in PBS samples compared to all other groups, Supplementary Figure 14d. No differences were found between all gels and SHAM samples. These findings corroborate the immune response expression previously explained. While being a delivery vehicle and protecting cells from the intrinsic immune response, hydrogels (and especially these IPN) also facilitate the entry of exogenous cells inside the layers of the retina with less Muller cell activation observed.

# Supplementary Data - Image processing algorithm

import cv2

import glob

import math

import matplotlib.pyplot as plt

import numpy as np

import json

from shapely.geometry import Point

from shapely.geometry.polygon import Polygon

import os

import pandas as pd

def make_frame_grid(frames, frames_info=None, num_cols=5, size=1.5, fontsize=8, save_path=None):

    num_lines = len(frames) // num_cols + 1

    h, w, _ = frames[0].shape

    ratio = h/w

    fig = plt.figure(figsize=(num_cols*size/ratio,num_lines*size))

    plt.tight_layout()

    for i, frame in enumerate(frames):

        ax1 = fig.add_subplot(num_lines, num_cols, i+1)

        ax1.set_yticklabels([])

        ax1.set_xticklabels([])

        if frames_info is not None:

            info = frames_info[i]

           # ax1.set_xlabel(info)

            ax1.set_title(info, fontdict={'fontsize': fontsize})

        ax1.imshow(frame)

        plt.subplots_adjust(wspace=.001, hspace=.3 if frames_info is not None else 0.001)

    if save_path is not None:

        plt.savefig(save_path,bbox_inches = 'tight',pad_inches = 0)

    else:

        plt.show()

    plt.close()

json_files = ['labels_gtn copy.json']#, 'labels_ipn75.json', 'labels_pbs.json', 'layers_ipn50.json']

by_file = {}

by2_file = {}

for file in json_files:

    with open(file, 'r') as f:

        lines = json.load(f)

    for key, value in lines.items():

        regions = value['regions']

        num_regions = len(regions)

        polygon_per_regions = {}

        points_per_regions = {}

        for _, region in regions.items():

            points_x = region['shape_attributes']['all_points_x']

            points_y = region['shape_attributes']['all_points_y']

            points = []

            for x, y in zip(points_x, points_y):

                points.append((x,y))

            region_name = region['region_attributes']['label']

            polygon_per_regions[region_name] = Polygon(points)

            points_per_regions[region_name]  = points

        by_file[key] = polygon_per_regions

def polygon_orientation(points, debug=False):

    i=0.001

    cv2_contour = []

    for p in points:

        cv2_contour.append([p])

    while len(cv2_contour)<5:

        cv2_contour.append([p])

        i=i+0.001

    cv2_contour = np.array(cv2_contour, dtype=np.int32)

    ellipse = cv2.fitEllipse(cv2_contour)

    if debug:

        canvas = np.ones_like(img)*255

        canvas = cv2.drawContours(canvas, [cv2_contour], -1, (0,255,0), 3)

        cv2.ellipse(canvas, ellipse, (0,255,255))

        plt.imshow(canvas)

    return ellipse

def get_centers(green):

    GREEN_THRESHOLD = 55 # To select only the really green cells

    BLUR_SIZE = 1 # To smooth the image

    CLOSING_SIZE = 1 # To remove the holes inside cells

    OPENING_SIZE = 2 # To remove the noise (small dots) around the cell

    blurred = cv2.blur(green, (BLUR_SIZE, BLUR_SIZE))

    thresholded = np.array(blurred > GREEN_THRESHOLD, dtype=np.uint8) * 255

    kernel = np.ones((CLOSING_SIZE, CLOSING_SIZE), np.uint8)

    imask = cv2.morphologyEx(thresholded, cv2.MORPH_CLOSE, kernel)

    kernel = np.ones((OPENING_SIZE, OPENING_SIZE), np.uint8)

    imask = cv2.morphologyEx(imask, cv2.MORPH_OPEN, kernel)

    return imask

GREEN_THRESHOLD = 30 # To select only the really green cells

BLUR_SIZE = 5 # To smooth the image

# Explainations: https://opencv-python-tutroals.readthedocs.io/en/latest/py_tutorials/py_imgproc/py_morphological_ops/py_morphological_ops.html

DILATATION_SIZE = 10

CLOSING_SIZE = 10 # To remove the holes inside cells

OPENING_SIZE = 5 # To remove the noise (small dots) around the cell

column_names = ["layer", "file", "group", "center", "size", "center", "axes", "angle cell","angle layer"]

result_lines = []

for file, info in by_file.items():

    group = file.split('_')[0]

    file_id = file.replace('.png', '')

    path = os.path.join('data', file_id, file_id + '.tif')

    img = cv2.imread(path)[:,:,::-1]

    green = cv2.imread(path.replace('shot4', 'shot2'))[:,:,1]

    centers = get_centers(green)

    blurred = cv2.blur(green, (BLUR_SIZE, BLUR_SIZE))

    thresholded = np.array(blurred > GREEN_THRESHOLD, dtype=np.uint8) * 255

    kernel = np.ones((DILATATION_SIZE,DILATATION_SIZE),np.uint8)

    eroded = cv2.dilate(thresholded,kernel,iterations = 1)

    kernel = np.ones((CLOSING_SIZE, CLOSING_SIZE), np.uint8)

    imask = cv2.morphologyEx(thresholded, cv2.MORPH_CLOSE, kernel)

    kernel = np.ones((OPENING_SIZE, OPENING_SIZE), np.uint8)

    imask = cv2.morphologyEx(imask, cv2.MORPH_OPEN, kernel)

    connectivity = 4  # You need to choose 4 or 8 for connectivity type

    ret, markers, stats, centroids = cv2.connectedComponentsWithStats(eroded, connectivity , cv2.CV_32S)

    # remove components without any center in them + save their center

    clean_centroids = []

    for component_id, centroid in enumerate(centroids):

        component_mask = np.where(markers == component_id)

        masked_centers = centers[component_mask]

        masked_eroded = np.array(markers == component_id, dtype=np.uint8)

#         print(ellipse)

        if np.sum(masked_centers) == 0:

            markers[component_mask] = 0

        else:

            area = len(component_mask[0])

            contours, hierarchy = cv2.findContours(masked_eroded, cv2.RETR_EXTERNAL,cv2.CHAIN_APPROX_NONE)

            if len(contours[0]) <= 5:

                print("ERROR WITH CONTOUR for " + file)

            else:

                ellipse = cv2.fitEllipse(contours[0])

                clean_centroids.append((centroid, area / (img.shape[0] * img.shape[1]), ellipse))

    composed = np.zeros_like(img)

    Polylayers = np.zeros_like(img,dtype=np.uint8)

    no_markers = markers == 0

    markers = markers * 4 + 10

    markers[no_markers] = 0

    composed[:,:, 0] = markers

    composed[:,:, 1] = centers

    # Draw the centers

    for (centroid, area, ellipse) in clean_centroids:

        center = (int(centroid[0]), int(centroid[1]))

        cv2.circle(composed, center, 10, (255, 0, 0), 2)

        cv2.ellipse(composed, ellipse,(0,255,255))

        single_point = Point(center[0], center[1])

        for region_id, polygon in info.items():

            ## Calculation of layer orientation with averaged of each side of polygon (NOT GOOD) - beter do with ellipse

            a=list(polygon.boundary.coords)

            cv2.polylines(Polylayers, np.int32([a]), 1, (255,255,255),thickness=2) # Polylayers is the 3rd picture with the layer and if possible the ellipse of the layer

            Sum1=0

            Sum2=0

            L=np.zeros(len(a)-1)

            Acos=np.zeros(len(a)-1)

            for i in range(0, len(a)-1):

                L[i]=math.sqrt(math.pow(a[i][0]-a[i+1][0],2)+math.pow(a[i][1]-a[i+1][1],2))

                Acos[i]=math.fmod(180*math.acos((a[i][0]-a[i+1][0])/L[i])/math.pi,90)

                Sum1=Sum1+(L[i]*Acos[i])

                Sum2=Sum2+L[i]

            layerangle=Sum1/Sum2

            ## End of calculation of orientation (You can get rid of this if ellipse gves better results)

            if polygon.contains(single_point):

                center, axes, angle = ellipse

                Grandaxe=axes[1]

                #angle=math.fmod(angle,90)

                polygon_ellipse = polygon_orientation(list(polygon.boundary.coords))

                cv2.ellipse(Polylayers, polygon_ellipse,(0,0,255))

                _, poly_axes, poly_angle = polygon_ellipse

                result_lines.append([region_id, file, group, centroid, area, center, Grandaxe, angle, poly_angle])

                print("center {} is in polygon for region {} with {} and {}".format(centroid, region_id, Grandaxe,poly_angle))

    make_frame_grid([img, composed,Polylayers], num_cols=3, size=70, save_path="ipn6.jpeg")

df = pd.DataFrame(result_lines, columns=column_names)

df.to_excel('results.xls')

# Supplementary References

1 Riaz, T. et al*.* FTIR analysis of natural and synthetic collagen. *Applied Spectroscopy Reviews* **53**, 703-746 (2018).

2 Oliveira, S. et al. Production and characterization of bacterial cellulose membranes with hyaluronic acid from chicken comb. *International Journal of Biological Macromolecules* **97** (2017).

3 Dromel, P. C. et al. Injectable gelatin hydroxyphenyl propionic acid hydrogel protects human retinal progenitor cells (hRPCs) from shear stress applied during small-bore needle injection. *Applied Materials Today* **19**, 100602 (2020).

4 Bae, J. W., Choi, J. H., Lee, Y. & Park, K. D. Horseradish peroxidase-catalysed in situ-forming hydrogels for tissue-engineering applications. *Journal of Tissue Engineering and Regenerative Medicine* **9**, 1225-1232 (2015).

5 Yongming, C., Biddell, K., Aiying, S., Relue, P. A. & Johnson, J. D. in *Proceedings of the First Joint BMES/EMBS Conference. 1999 IEEE Engineering in Medicine and Biology 21st Annual Conference and the 1999 Annual Fall Meeting of the Biomedical Engineering Society (Cat. N.* 819 vol.812).

6 Sluch, V. M. et al. Differentiation of human ESCs to retinal ganglion cells using a CRISPR engineered reporter cell line. *Scientific Reports* **5**, 16595 (2015).

7 Sluch, V. M. et al*.* Enhanced Stem Cell Differentiation and Immunopurification of Genome Engineered Human Retinal Ganglion Cells. *Stem cells translational medicine* **6**, 1972-1986 (2017).

8 Lim, T. C. et al. Hydrogel-Based Therapy for Brain Repair After Intracerebral Hemorrhage. *Translational Stroke Research* **11**, 412-417 (2020).

9 Schwartz, D. M., Shuster, S., Jumper, M. D., Chang, A. & Stern, R. Human vitreous hyaluronidase: isolation and characterization. *Current Eye Research* **15**, 1156-1162 (1996).

10 van Deemter, M. et al. Enzymatic breakdown of type II collagen in the human vitreous. *Invest Ophthalmol Vis Sci* **50**, 4552-4560 (2009).

11 Danias, J. et al. Cytoarchitecture of the Retinal Ganglion Cells in the Rat. *Investigative Ophthalmology & Visual Science* **43**, 587-594 (2002).

12 Rockhill, R. L., Daly, F. J., MacNeil, M. A., Brown, S. P. & Masland, R. H. The diversity of ganglion cells in a mammalian retina. *Journal of Neuroscience* **22**, 3831-3843 (2002).

13 Kreft, M., Milisav, I., Potokar, M. & Zorec, R. Automated high through-put colocalization analysis of multichannel confocal images. *Computer Methods and Programs in Biomedicine* **74**, 63-67 (2004).

14 Xu, H., Chen, M., Mayer, E. J., Forrester, J. V. & Dick, A. D. Turnover of resident retinal microglia in the normal adult mouse. *Glia* **55**, 1189-1198 (2007).
