# Supplementary material for: A bioinspired gelatin-hyaluronic acid-based hybrid interpenetrating network for the enhancement of retinal ganglion cells replacement therapy
Source: NPJ Regen Med. 2021 Dec 20;6:85. doi: 10.1038/s41536-021-00195-3 (PMC8688498; doi:10.1038/s41536-021-00195-3)
Supplement: Supplementary file 2 — Reporting Summary [file 41536_2021_195_MOESM2_ESM.pdf]

nature portfolio

Corresponding author(s): Michael Young

Last updated by author(s): Oct 12, 2021

Reporting Summary

Nature Portfolio wishes to improve the reproducibility of the work that we publish. This form provides structure for consistency and transparency in reporting. For further information on Nature Portfolio policies, see our Editorial Policies and the Editorial Policy Checklist.

Statistics

For all statistical analyses, confirm that the following items are present in the figure legend, table legend, main text, or Methods section.

n/a	Confirmed	
☐	☒	The exact sample size (n) for each experimental group/condition, given as a discrete number and unit of measurement
☐	☒	A statement on whether measurements were taken from distinct samples or whether the same sample was measured repeatedly
☐	☒	The statistical test(s) used AND whether they are one- or two-sided<br>*Only common tests should be described solely by name; describe more complex techniques in the Methods section.*
☐	☒	A description of all covariates tested
☐	☒	A description of any assumptions or corrections, such as tests of normality and adjustment for multiple comparisons
☐	☒	A full description of the statistical parameters including central tendency (e.g. means) or other basic estimates (e.g. regression coefficient) AND variation (e.g. standard deviation) or associated estimates of uncertainty (e.g. confidence intervals)
☒	☐	For null hypothesis testing, the test statistic (e.g. F, t, r) with confidence intervals, effect sizes, degrees of freedom and P value noted<br>*Give P values as exact values whenever suitable.*
☒	☐	For Bayesian analysis, information on the choice of priors and Markov chain Monte Carlo settings
☒	☐	For hierarchical and complex designs, identification of the appropriate level for tests and full reporting of outcomes
☒	☐	Estimates of effect sizes (e.g. Cohen's d, Pearson's r), indicating how they were calculated

Our web collection on statistics for biologists contains articles on many of the points above.

Software and code

Policy information about availability of computer code

Data collection	No software was used
Data analysis	Graphpad Prism, MATLAB and MAKESENSE.AI

For manuscripts utilizing custom algorithms or software that are central to the research but not yet described in published literature, software must be made available to editors and reviewers. We strongly encourage code deposition in a community repository (e.g. GitHub). See the Nature Portfolio guidelines for submitting code & software for further information.

Data

Policy information about availability of data

All manuscripts must include a data availability statement. This statement should provide the following information, where applicable:

- Accession codes, unique identifiers, or web links for publicly available datasets
- A description of any restrictions on data availability
- For clinical datasets or third party data, please ensure that the statement adheres to our policy

The data that supports the findings of this study are available from the corresponding author upon reasonable request.

Field-specific reporting

Please select the one below that is the best fit for your research. If you are not sure, read the appropriate sections before making your selection.

☒ Life sciences ☐ Behavioural & social sciences ☐ Ecological, evolutionary & environmental sciences

For a reference copy of the document with all sections, see nature.com/documents/nr-reporting-summary-flat.pdf

Life sciences study design

All studies must disclose on these points even when the disclosure is negative.

Sample size	Sample size was mostly n=15 and experiments were set up in triplicates. We did power analysis and this was the ideal number for sample size.
Data exclusions	NO
Replication	All experiments in-vitro were set up in triplicates and in-vivo each group was set up in n=5. All replication were successful
Randomization	Animals were randomly divided in group of 5. The groups were all female and injected on the same day.
Blinding	The experiment was performed as double blind study as the surgeon was unaware of the material injected. For all data analysis with our proprietary image processing algorithms that the images were assigned to the group post analysis.

Reporting for specific materials, systems and methods

We require information from authors about some types of materials, experimental systems and methods used in many studies. Here, indicate whether each material, system or method listed is relevant to your study. If you are not sure if a list item applies to your research, read the appropriate section before selecting a response.

Materials & experimental systems

n/a	Involved in the study	
☐	☒	Antibodies
☐	☒	Eukaryotic cell lines
☒	☐	Palaeontology and archaeology
☐	☒	Animals and other organisms
☒	☐	Human research participants
☒	☐	Clinical data
☒	☐	Dual use research of concern

Methods

n/a	Involved in the study	
☒	☐	ChIP-seq
☐	☒	Flow cytometry
☒	☐	MRI-based neuroimaging

Antibodies

Antibodies used	STEM121 monoclonal no: Y40410 TakaraBio, DAPI no: 564907 BD Biosciences, Anti Cas9 mouse monoclonal no: 7A9-3A3 Santa Cruz, Anit-Cmyc monoclonal mouse no: 551101 BD Biosciences, Brn3a polyclonal no: BS-3669R ThermoFischer, Anti-IBA1 goat polyclonal no: ab5076 Abcam, Anti-Ki67 mouse monoclonal no: sc-23900 Santa Cruz, Anti-RBPMS polyclonal no: ABN1376 EMD Milipore, Thy1.1 anti-human monoclonal no: REA897 Miltenyi Biotec, Anti-NeuN rabbit monoclonal no: EPR12763 Abcam, Anti-Oct4 clone 40 no: 611203 BD Biosciences, CD45 rabbit polyclonal no: ab10558 Abcam, Anti-GFAP (STEM123) mouse monoclonal no: Y40420 TakaraBio, CD11b anti-rat no: REA325 Miltenyi Biotec, CD68 anti-rat no: REA237 Miltenyi Biotec, Rabbit IgG monoclonal no: ab125938 Abcam, Mouse IgG no: ab37355 Abcam.
Validation	Miltenyi Biotec: REAfinity antibodies ares tested and validates with reproducibility and consistency (pure antibodies, lot to lot consistency), specificity (epitope competition assay, genome validation) and sensitivity (functional testing, performance comparison and fixation compatibility).<br>Abcam: Validation performed with IHC and ICC, Mass spec, KO cell lines, protein assay, protein expression profile, cell treatment siRNA knockdown.<br>BD Biosciences: Testing on combination of primary cells, cell line, transfectant cells. Multiplexing with additional antibodies.<br>TakaraBio: Testing in our laboratory on different primary cell line was performed to obtain the correct concentration for optimal expression.<br>Santa Cruz: Testing in our laboratory on different primary cell line was performed to obtain the correct concentration for optimal expression.<br>EMD Milipore: Flow cytometry, IHC, ICC, primary cell lines, 3D structures in proteins.

Eukaryotic cell lines

Policy information about cell lines

Cell line source(s)	hRGC isolated and purified in Dr. Donald Zack laboratory
Authentication	Authentication performed with flow cytometry. https://www.nature.com/articles/srep16595
Mycoplasma contamination	Not tested for mycoplasma contamination
Commonly misidentified lines (See ICLAC register)	None

Animals and other organisms

Policy information about studies involving animals; ARRIVE guidelines recommended for reporting animal research

Laboratory animals	Long Evans rats, female, 12 weeks
Wild animals	N/A
Field-collected samples	N/A
Ethics oversight	Schepens Eye Research institute Animal Facility and Association for Research in vision Ophthalmology Statement for the use of animals in ophthalmic and vision research.

Note that full information on the approval of the study protocol must also be provided in the manuscript.

Flow Cytometry

Plots

Confirm that:

☒ The axis labels state the marker and fluorochrome used (e.g. CD4-FITC).

☒ The axis scales are clearly visible. Include numbers along axes only for bottom left plot of group (a 'group' is an analysis of identical markers).

☒ All plots are contour plots with outliers or pseudocolor plots.

☒ A numerical value for number of cells or percentage (with statistics) is provided.

Methodology

Sample preparation	hRGC [(3 x 105 cells/mL in PBS or in 1 mL of Gtn-HPA, IPN75 and IPN50 hydrogels, in 6-well plates (3.5 cm diameter, polystyrene, flat bottom, sterile, fisher scientific)] were maintained in PBS for 5 days (replicating the in vivo conditions). 1000 U/mL collagenase-type IV (Invitrogen) was then added to degrade the Gtn-HPA hydrogels and 500 U/mL hyaluronidase type I-S (Sigma-Aldrich) to degrade the HA-Tyr part of the IPN; after 20 minutes of incubation, Gtn-HPA, IPN75 and IPN50 gels were fully dissolved. Samples were centrifuged, and hRGC were harvested. The phenotype of hRGC was analyzed using flow cytometry with the MACSQuant flow cytometer (Miltenyi, San Diego). hRGC, from the 4 different conditions—in PBS, in Gtn-HPA, IPN75 and IPN50 were collected and fixed with a Perm/Fix buffer (BD Biosciences) at 4 C for 15 min. Cells were then washed in a wash buffer (BD Biosciences) and incubated, at room temperature, in a blocking buffer (Pharmingen staining buffer with 2% goat serum) for 30 min. Blocked cells were seeded onto a flat bottom 96-well plate (treated, sterile, polystyrene, Thomas Scientific) and labeled overnight at room temperature with the following conjugated primary antibodies: Brn3a-FITC, RBPMS-APC, Thy1.1-APC (ganglion cell marker), Caspase9-FITC (apoptosis marker), Ki67-FITC (proliferation marker),� Cmyc-FITC, Oct4-APC (stemness markers) and NeuN-APC (neuronal marker). Primary antibodies were diluted in 200 uL of antibody buffer (TBS, 0.3% Triton X-100 and 1% goat serum). After overnight incubation cells were washed three times for 15 min, and incubated in secondary antibodies and left at room temperature for 3h; secondary antibodies (goat-derived anti-rabbit and anti-mouse, DAPI-VioBlue) were diluted 1:200 in antibody buffer (Jackson Immunoresearch Laboratory) .
Instrument	MACSQuant flow cytometer 10 (Miltenyi, San Diego),
Software	The results were analyzed using the MACSQuantify software (https://www.miltenyibiotec.com)
Cell population abundance	Light scatter and fluorescence signals from each sample were measured using the MACSQuant (Miltenyi Biotech, Germany) flow cytometer (2 x 10^5 events were recorded).

Gating strategy

For each primary antibody the FSC/SSC cell population was gated (postive in the center of the graph), then the FSC-A vs FSC-H was gating along the linear line (including only single cells) and finally the DAPI-positive single cell population was gated (higher than 10^2). The ratio of positive cells in the gated population was estimated in comparison with blank (gate put at 0%) and species-specific isotype controls (gate placed at 2.5%) being above 10^1 in most cases.

☒ Tick this box to confirm that a figure exemplifying the gating strategy is provided in the Supplementary Information.
